# Single‐Cell Profiling Identifies *SLC2A5*‐Mediated Fructose Metabolism as a Vulnerability in Primary CNS Lymphoma

**DOI:** 10.1002/advs.76316

**Published:** 2026-06-26

**Authors:** Qiaoli Wu, Qianru Zhang, Wenqiang Yan, Lu Sun, Yiran Cui, Bolin Liu, Guoqing Han, Feng Zhang, Ke Pu, Mingchao Zhang, Xiaobing Zhang, Qingguo Li, Gang An, Yuxuan Liu

**Affiliations:** ^1^ Huanhu Hospital Affiliated to Tianjin Medical University Tianjin Medical University Tianjin China; ^2^ Tianjin Key Laboratory of Cerebral Blood Flow Reconstruction and Head and Neck Tumor New Technology Translation Tianjin Neurosurgical Institute Tianjin Huanhu Hospital Tianjin China; ^3^ State Key Laboratory of Experimental Hematology National Clinical Research Center for Blood Diseases Haihe Laboratory of Cell Ecosystem Institute of Hematology & Blood Diseases Hospital Chinese Academy of Medical Sciences & Peking Union Medical College Tianjin China; ^4^ Tianjin Institutes of Health Science Tianjin China; ^5^ Department of Neurosurgery Tianjin Huanhu Hospital Tianjin China

**Keywords:** biology, cancer research, fructose, lymphoma, primary central nervous system lymphoma, single‐cell analysis, tumor microenvironment

## Abstract

Primary central nervous system lymphoma (PCNSL) exhibits distinct molecular features and a unique tumor microenvironment (TME) characterized by hypoxia and reduced cerebrospinal fluid glucose levels. However, the extent to which the PCNSL TME shapes the metabolic and functional states of tumor and non‐tumor microenvironment cells remains largely unexplored. Utilizing single‐cell multi‐omic approaches, we systematically dissected tumor‐TME interactions in PCNSL and showed that glucose deprivation within the TME leads to enhanced *SLC2A5*‐mediated fructose metabolism in tumor cells and contributes to T cell dysfunction. Furthermore, hypoxia within the TME induces *SLC2A5* expression in tumor‐supportive macrophages through HIF‐dependent transcriptional regulation, establishing *SLC2A5* and its associated fructose metabolism as potential metabolic vulnerabilities in both tumor cells and tumor‐supportive macrophages. In vitro and in vivo functional assays further demonstrated that genetic and pharmacologic inhibition of *SLC2A5*‐mediated fructose uptake markedly suppressed lymphoma growth. Collectively, our study uncovers a novel potential metabolic liability targeting tumor–TME interactions in PCNSL.

## Graphical abstract

Glucose deprivation in the primary CNS lymphoma (PCNSL) tumor microenvironment drives *SLC2A5* (encoding GLUT5)‐dependent fructose metabolism in tumor cells, while hypoxia induces HIF‐mediated *SLC2A5* expression in tumor‐supportive macrophages, revealing *SLC2A5*‐driven fructose utilization as a shared and targetable metabolic vulnerability across malignant and microenvironmental compartments.

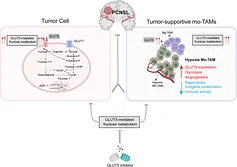

## 1 Introduction

Primary central nervous system lymphoma (PCNSL), a rare subtype of diffuse large B cell lymphoma (DLBCL), is an extranodal non‐Hodgkin's lymphoma that arises exclusively in the central nervous system (CNS), with the brain parenchyma being the most frequently affected site. PCNSL is associated with a poorer prognosis compared to systemic DLBCL [1]. Although high‐dose methotrexate (HD‐MTX)‐based regimens, in combination with consolidation therapies such as high‐dose chemotherapy followed by autologous stem cell transplantation, whole‐brain radiotherapy (WBRT), and/or non‐myeloablative chemotherapy, have demonstrated efficacy in fit PCNSL patients, a substantial proportion fail to achieve a response to HD‐MTX‐based chemotherapy (15%–25%) or experience disease relapse following an initial response (25%–50%) [2].

PCNSL exhibits distinct genomic alterations and transcriptional profiles compared to systemic DLBCL. For example, although PCNSL predominantly aligns with the MYD88/CD79B‐mutated (MCD) genetic subtype of DLBCL, approximately 10% to 23% of PCNSL samples harbor mutations in *B2M*, *GNA13*, *ITPKB*, *P2RY8*, and *PABPC1*, whereas fewer than 5.1% of MCD DLBCL cases exhibit these alterations [3, 4]. PCNSL also displays significantly more frequent focal deletions at the 6p21 locus compared to systemic DLBCL [4]. Moreover, at the transcriptomic level, PCNSL is characterized by a distinct gene expression pattern that clearly distinguishes it from systemic DLBCL [4].

The tumor microenvironment (TME) of PCNSL also exhibits unique immunological and metabolic features. Comparative analyses between PCNSL and systemic DLBCL have shown that the TME of PCNSL is more immunosuppressive, characterized by reduced infiltration of effector T cells and elevated T cell exhaustion scores [5, 6, 7, 8]. In addition, CNS lymphoma is recognized as one of the diseases resulting in low cerebrospinal fluid (CSF) glucose levels. A study characterizing malignant lymphoma with CNS involvement found that 54% of patients with CNS lymphoma exhibited CSF glucose levels below 50 mg dL^−1^, and 19% had levels under 15 mg dL^−1^ [9]. Consistently, another study on leptomeningeal lymphoma reported that 54% of patients exhibited hypoglycorrhachia, defined as CSF glucose <50 mg dL^−1^ [10]. Moreover, the expression of hypoxia‐associated proteins in PCNSL suggests the presence of a hypoxic tumor microenvironment [11]. However, the extent to which the PCNSL microenvironment affects both malignant cells and non‐malignant TME cells, as well as how the unique molecular features of PCNSL shape infiltrating immune cells, remains largely unexplored.

Here, we dissected the interactions between tumor cells and their microenvironment in PCNSL using single‐cell RNA‐seq (scRNA‐seq) in combination with single‐cell B‐cell receptor sequencing (scBCR‐seq). Our study demonstrates that the PCNSL TME, characterized by glucose deprivation and hypoxia, drives metabolic reprogramming in both tumor and microenvironment cells, leading to changes in cellular composition and function that support PCNSL pathogenesis. We further show that molecular features intrinsic to PCNSL, in turn, modulate infiltrating immune cells. Collectively, our study elucidates the molecular and cellular mechanisms underlying PCNSL development and identifies a novel potential metabolic vulnerability targeting the tumor‐TME interactions in PCNSL.

## 2 Results

### 2.1 Single‐Cell Landscape of PCNSL and Non‐Tumor Controls

We performed scRNA‐seq together with scBCR‐seq on PCNSL tumor specimens and three control groups obtained from peripheral blood (PB), tumor‐adjacent tissues, and surgical resections from individuals with traumatic brain injuries (Figure 1A; Figure S1 and Table S1). After filtering out low‐quality cells, we obtained 140844 single cells in total. Cell types in PCNSL tumors and control samples were identified and annotated based on the expression of canonical gene markers (Figure 1B–D; Figure S2A–L; Methods).

**FIGURE 1:**
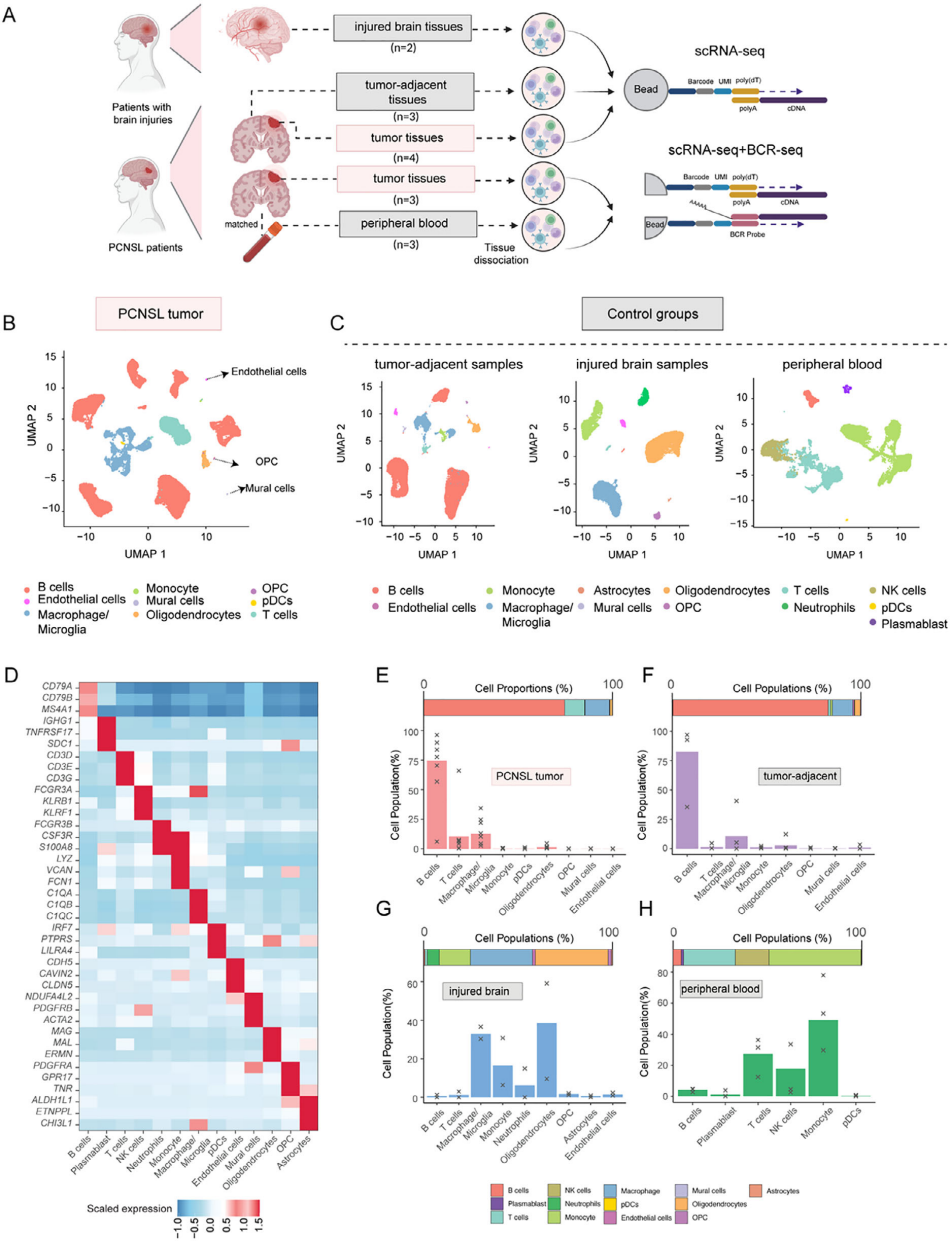
Single‐cell landscape of PCNSL and control samples. (A) Schematic overview of the experimental design, including sample collection and sequencing strategy. (B) Uniform manifold approximation and projection (UMAP) visualization of single‐cell transcriptomes from 7 primary central nervous system lymphoma (PCNSL) samples. Each dot represents a single cell, colored by cell types. (C) UMAP projection of single‐cell transcriptomes from control samples, including tumor‐adjacent tissues (left) from 3 patients, injured brain tissues (middle) from 2 patients, and peripheral blood (PB) (right) from 3 patients. Each point represents an individual cell and is colored according to its assigned cell type. (D) Heatmap showing normalized expression of selected cell‐type specific genes, ordered by annotated cell types. (E–H) Distributions of cell types in PCNSL tumor samples (*n* = 7) (E), tumor‐adjacent samples (*n* = 3) (F), injured brain samples (*n* = 2) (right), and PB (*n* = 3) (H). Bar heights indicate mean frequencies of different patients; crosses (×) represent values for individual patients. The horizontal bars depict the overall proportion of each cell type across all patients within a sample group.

Across 58755 high‐quality cells profiled from seven PCNSL tumor samples, B cells (74.64%), T cells (10.65%), and macrophages/microglia (12.89%) constituted the predominant cell populations, whereas the remaining cells comprised minor, distinct populations, including monocytes, endothelial cells, mural cells, oligodendrocytes, oligodendrocyte progenitor cells (OPCs), and plasmacytoid dendritic cells (pDCs) (collectively 1.82%) (Figure 1E). The cellular composition of tumor‐adjacent samples (total 40802 cells from three patients) closely resembled that of PCNSL tumors, with B cells and macrophages/microglia representing the most abundant populations (82.57% and 10.79%, respectively), while other cell types (e.g., T cells, monocytes, oligodendrocytes and others) collectively accounted for a smaller fraction (6.64%) (Figure 1F). In injured brain samples (17910 cells from two patients), the baseline cellular landscape was dominated by myeloid populations, including macrophages/microglia (32.99%), monocytes (16.53%), and neutrophils (6.23%), along with oligodendrocytes (38.55%) (Figure 1G). In contrast, B cells, T cells, OPCs, astrocytes, and endothelial cells were relatively scarce, collectively accounting for only 5.70%. Among 23377 high‐quality PB cells from three patients, T cells (27.33%), NK cells (17.79%), and monocytes (49.07%) represented the major populations, whereas B cells, plasmablasts, and pDCs were present at comparatively low frequencies (5.81%) (Figure 1H).

### 2.2 Characterization of B Cells in PCNSL and Control Samples

We first investigated B cells across tumor (*n* = 7), tumor‐adjacent (*n* = 3), and PB (*n* = 3) samples. Uniform manifold approximation and projection (UMAP) visualization revealed that B cells from PCNSL tumor and tumor‐adjacent tissues were highly patient‐specific, whereas PB B cells exhibited similar gene expression profiles across patients and clustered together (Figure 2A). Shannon entropy analysis further demonstrated increased inter‐patient heterogeneity (i.e., lower entropy) in tumor and tumor‐adjacent B cells compared with PB B cells (Figure 2B). We next calculated the κ/λ light chain ratio of B cells across the three groups, a criterion commonly used to distinguish malignant from non‐malignant B cells. We found that non‐malignant PB B‐cell cluster contained both κ‐ and λ‐expressing B cells, whereas B cells from PCNSL tumors and tumor‐adjacent tissues exclusively expressed the κ light chain, suggesting that B cell population in tumor‐adjacent tissues may also display malignant features (Figure 2C,D). We further performed single‐cell copy number variation (CNV) analysis on tumor and tumor‐adjacent B cells using inferCNV. This analysis revealed that B cells from tumor‐adjacent tissues in all three patients exhibited altered CNVs, including canonical PCNSL‐associated lesions such as losses in 6p21 and 19p13 and gains in 18q21 (Figure S3A,D,G), further supporting their malignant nature. Notably, tumor and tumor‐adjacent B cells from patient 1 displayed distinct CNV patterns and segregated into separate clusters in UMAP space, whereas those from patient 2 and patient 3 exhibited similar CNV patterns and were clustered together (Figure 2A; Figure S3A–I).

**FIGURE 2:**
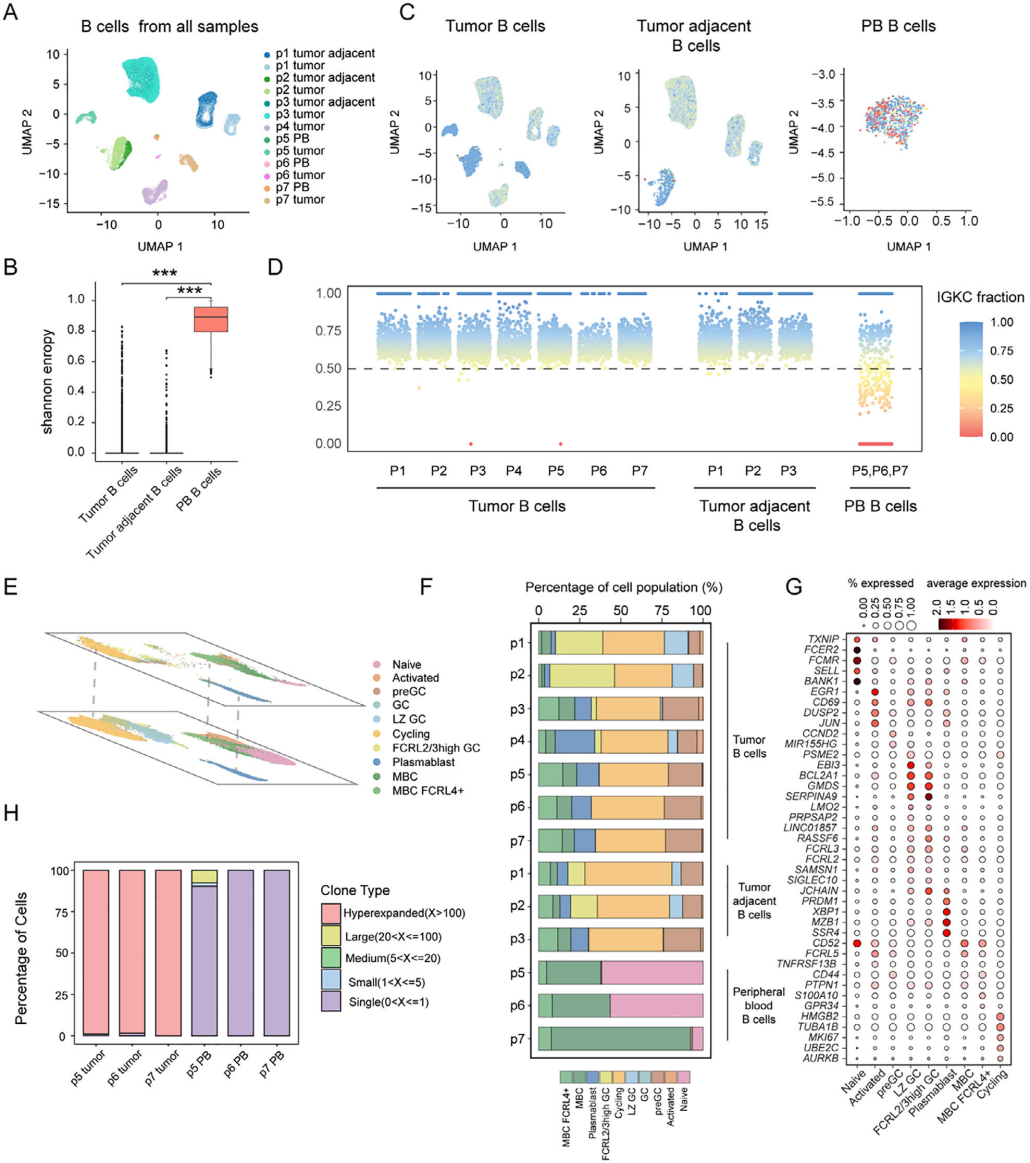
Characterization of B cells in PCNSL and control samples. (A) UMAP visualization of B cells derived from PCNSL, tumor‐adjacent tissues and PB, colored by sample origins and patient identities. (B) Inter‐patient heterogeneity of B cells derived from PCNSL, tumor‐adjacent tissues, and PB, quantified by Shannon entropy. (C) UMAP visualization of κ/λ light chain ratios in B cells from PB, PCNSL, and tumor‐ adjacent tissues. The κ/λ ratio for each cell was calculated based on the single‐cell gene expression levels of *IGKC* and *IGLC2*, which encode the constant regions of the κ and λ light chains, respectively. B cells with an *IGKC* fraction greater than 0.5 were designated as κ‐ expressing B cells, and those with an *IGKC* fraction less than 0.5 are classified as λ‐expressing B cells. Each dot represents a single cell, with color indicating *IGKC* fraction. (D) Distribution of κ/λ ratios in B cells from PB, PCNSL, and tumor‐adjacent tissues. Each dot represents a single cell, colored by *IGKC* fraction. To make cell numbers comparable across different samples, all cells from PB group and patient 6 in the PCNSL group are included, and 1000 randomly subsampled cells from each of the remaining samples are displayed. (E) Projection of B cells from PCNSL, tumor‐adjacent tissues, and PB to an annotated tonsillar B cell reference. B cells were annotated based on the predefined subtypes from the reference dataset. (F) Proportions of B cell subtypes among total B cells in each patient sample. (G) Expression levels of selected marker genes across B cell subtypes. (H) Distribution of B cell clone types in PCNSL and PB samples from each patient. Clonotypes are categorized based on clone size: clones with >100 cells are defined as hyperexpanded, 21–100 as large, 6–20 as medium, 2–5 as small, and 1 as single‐cell clones.

Subsequently, we compared B cell states across the three groups. By mapping B cells from PCNSL, tumor‐adjacent, and PB samples to an annotated tonsillar B cell reference [12], diverse B cell states were identified (Figure 2E,F). Consistent with the germinal center (GC) origin of PCNSL, B cells from PCNSL tumors were predominantly projected to various differentiation stages of GC B cells and expressed GC B cell markers (Figure 2F,G) [13]. Tumor‐adjacent B cells exhibited a similar distribution to tumor samples, whereas PB B cells were primarily projected to naïve and memory B cells (Figure 2F). Non‐negative matrix factorization (NMF) analysis of malignant B cells identified six transcriptional metaprograms in PCNSL tumor B cells (Figure S4A). In line with the highly proliferative nature of PCNSL, as evidenced by a high Ki‐67 proliferative index [14], metaprogram associated with cell cycle and DNA replication (MP1 and MP2), which were predominantly expressed in cells mapped to the cycling B cell population, were detected in all seven patients (Figure S4A–C). Metaprogram MP3, which is linked to endoplasmic reticulum stress, was identified in five patients and predominantly expressed in cells projected to plasmablast cell population (Figure S4A–C). The plasmablast‐like signature has been reported to be associated with a worse prognosis in PCNSL [7].

To investigate the clonal origin of PCNSL, we performed scRNA‐seq coupled with scBCR‐seq on tumor and PB samples from three PCNSL patients. A total of 6246 B cells with CDR3 sequences detected in both the heavy and light chains were successfully matched to scRNA‐seq data. Clonal analysis revealed that tumor samples predominantly comprised hyperexpanded clones, whereas PB samples primarily contained unexpanded (single clone) cells, indicating that nearly all B cells in PCNSL tumor samples originate from a single clonal lineage (Figure 2H). Notably, the hyperexpanded clones identified in tumor samples were absent in PB, suggesting they are not blood‐derived (Table S2).

Collectively, these results demonstrate that PCNSL tumor‐adjacent tissues harbor a substantial population of B cells with malignant features, underscoring the importance of administering systematic chemotherapy and/or WBRT in PCNSL. Furthermore, tumor B cells originate from a common clonal lineage while exhibiting diverse transcriptional states.

### 2.3 Fructose Metabolism Is Upregulated in PCNSL

To identify malignant gene programs in PCNSL, we compared B cells from tumor samples with normal B cells from patient‐matched PB (*n* = 3). Genes involved in MHC class II‐mediated antigen processing and presentation (e.g., *HLA‐DRA*, *HLA‐DRB1*, *HLA‐DPA1*) were significantly downregulated in PCNSL (Figure 3A,B). This downregulation may be attributed to a recurrent genomic aberration in PCNSL involving the loss of chromosome 6p21.32, which harbors the HLA locus. Reduced expression of HLA class II genes may facilitate immune evasion of PCNSL tumor cells from CD8+ cytotoxic T cells. In addition to pathways known to be associated with aggressive B‐cell lymphoma, such as protein misfolding, cell cycle regulation, and B‐cell activation and differentiation, we observed marked upregulation of the fructose transporter *SLC2A5* and fructose metabolism pathway in PCNSL (Figure 3A,B).

**FIGURE 3:**
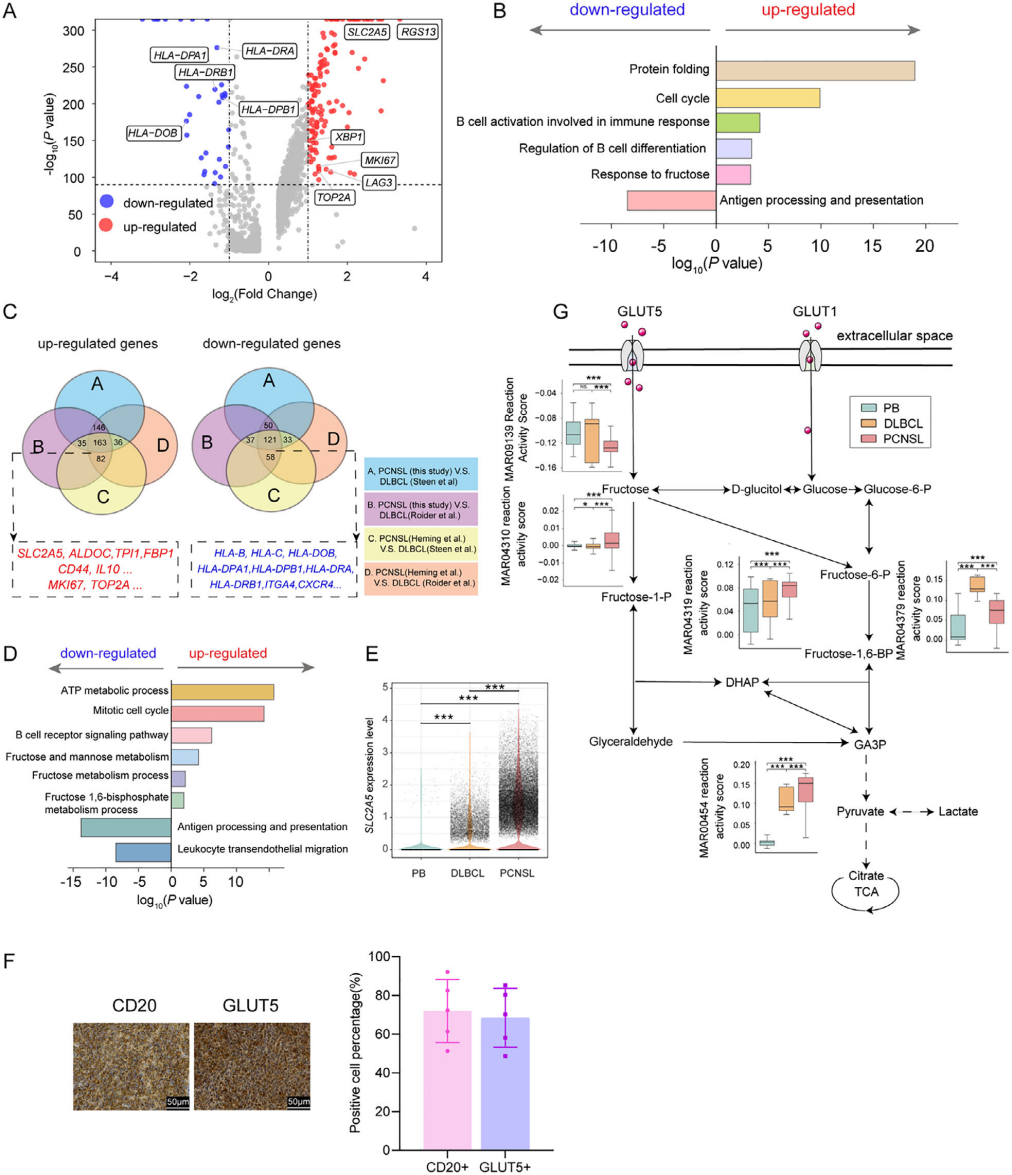
Fructose metabolism is upregulated in PCNSL. (A) Volcano plot showing differentially expressed genes in B cells between PCNSL tumor and PB samples. Genes with –log10(*p* value) > 90 and log2(fold change) > 1 are highlighted in red, and genes with –log10(*p* value) > 90 and log2(fold change) < –1 are highlighted in blue. (B) Representative pathways significantly upregulated or downregulated in B cells from PCNSL compared to PB. (C) Venn diagrams showing the overlap of upregulated (left) and downregulated (right) genes in B cells identified from comparisons between PCNSL (one dataset from this study and one from published data) and DLBCL (two publicly available datasets). (D) Representative pathways significantly upregulated and downregulated in B cells of PCNSL compared to DLBCL. (E) *SLC2A5* expression levels across PB, DLBCL and PCNSL samples, with *** indicating *p* < 0.001. (F) Immunohistochemical (IHC) staining of CD20 and GLUT5 showing that regions with high CD20 expression exhibit strong GLUT5 expression. Quantification of the percentages of CD20‐positive and GLUT5‐positive cells is shown on the right. Each dot represents an individual sample (*n* = 5), and bars indicate mean ± SD. Differences in the percentages of CD20‐positive cells and GLUT5‐positive cells across the five samples were accessed using Wilcoxon test (*p* = 0.69). (G) Inferred metabolic fluxes of fructose and glucose metabolism in B cells from PB (green), DLBCL (orange), and PCNSL (pink), estimated using METAFlux. The y‐axis represents flux scores calculated from METAFlux for each reaction. Differences in flux scores among groups were assessed using the Wilcoxon test, with *** indicating *p* < 0.001.

High‐grade lymphomas are characterized by elevated glucose uptake [15]. Brain ^18^F‐fluorodeoxyglucose (FDG)‐positron emission tomography imaging shows that PCNSL exhibits markedly elevated FDG uptake, indicative of enhanced glucose transport and glycolysis [16, 17]. However, physiological glucose levels in CSF are lower than those in serum, approximating 60% of serum glucose concentrations [18], and glucose levels in brain interstitial fluid are even lower [19]. In patients with CNS lymphoma, CSF glucose levels have been reported to drop below the physiological level of 50 mg/dL in more than half of cases [9, 10, 20]. Thus, high glucose demand of PCNSL cells, combined with limited glucose availability, may force these cells to utilize alternative nutrient source to meet their energy requirements. Accordingly, we investigated whether fructose metabolism serves as a key glucose alternative specific to PCNSL, facilitating its adaptation to low‐glucose conditions in the CNS microenvironment. To this end, we compared the transcriptional profiles of malignant B cells from PCNSL and systemic DLBCL. DLBCL tumor B‐cell transcriptomes were obtained from two publicly available scRNA‐seq datasets, including 4 DLBCL patients from Steen et al. and 3 DLBCL patients from Roider et al., whereas PCNSL tumor B‐cell expression profiles were derived from our study (7 PCNSL patients) and a previously published dataset (Heming et al.) comprising 5 PCNSL patient samples [8, 21, 22]. Similar to the comparison with PB, antigen processing and presentation genes (HLA class I and II) were downregulated in PCNSL (Figure 3C,D). Expression levels of *ITGA4* and *CXCR4*, involved in leukocyte transendothelial migration, were also reduced, possibly explaining why most PCNSL relapses remain confined to the central nervous system (Figure 3C) [23]. Compared to DLBCL, PCNSL exhibited more aggressive characteristics, with significant upregulation of ATP metabolism and mitotic cell cycle pathways (Figure 3D). Genes including *CD44* and *IL10* were also upregulated in PCNSL (Figure 3C). *CD44* may be involved in the homing of lymphoma cells into the CNS [24] and *IL10* was proposed as a diagnostic biomarker for PCNSL [25]. Similar to the comparison between PCNSL and PB, the fructose metabolism pathway and genes associated with fructose metabolism, such as *SLC2A5, ALDOC, TPI1, and FBP1* also showed upregulation in PCNSL compared to DLBCL, indicating that low glucose conditions in the CNS may drive PCNSL to utilize fructose metabolism as a crucial alternative energy source (Figure 3C–E). *SLC2A5* encodes the fructose transporter GLUT5. To confirm that GLUT5 is also highly expressed at the protein level in B cells from PCNSL clinical samples, we performed immunohistochemical (IHC) staining using antibodies against GLUT5 and CD20. We observed that regions with high CD20 expression also exhibited strong GLUT5 expression (Figure 3F; Figure S5A).

We further characterized glucose and fructose metabolism in B cells of PCNSL using METAFlux, a computational method that enables inference of metabolic fluxes from single‐cell transcriptomic data [26]. METAFlux analysis inferred increased fructose uptake and enhanced fructose‐related metabolic activity in PCNSL compared with DLBCL and PB (Figure 3G). For example, metabolic reactions specific to fructose metabolism, such as the conversion from fructose to fructose 1‐phosphate or fructose 6‐phosphate and the conversion from glyceraldehyde to glyceraldehyde 3‐phosphate (GA3P), displayed higher METAFlux‐inferred flux scores in PCNSL than in DLBCL and PB (Figure 3G). Conversely, for some reactions shared by both glucose and fructose metabolism, such as the conversion between fructose 6‐phosphate and fructose 1,6‐bisphosphate, METAFlux inferred higher activity scores in DLBCL compared with PCNSL (Figure 3G).

Given that all PCNSL samples included in this study were classified as the non‐germinal center B‐cell‐like (GCB) subtype (Table S1), we sought to exclude the possibility that the observed differences between PCNSL and DLBCL reflected broader biological distinctions between activated B‐cell‐like (ABC)‐DLBCL and GCB‐DLBCL. To this end, we further stratified DLBCL patients into ABC‐DLBCL and GCB‐DLBCL subtypes based on their diagnoses reported in the original studies [21, 22]. As expected, genes previously reported to be upregulated in ABC‐DLBCL, including *SH3BP5*, *IL16*, *IRF4*, *CCND2*, *ENTPD1*, *FUT8*, *PTPN1*, and *ETV6*, showed significant upregulation in B cells from ABC‐DLBCL patients, whereas the GCB‐DLBCL marker *MME* was more highly expressed in B cells from GCB‐DLBCL patients (Figure S5B) [27]. Comparative analyses of single‐cell transcriptomic profiles of B cells across PCNSL, GCB‐DLBCL and ABC‐DLBCL revealed that *SLC2A5* expression was consistently elevated in PCNSL relative to both GCB‐DLBCL and ABC‐DLBCL (Figure S5C–E). Furthermore, METAFlux analyses demonstrated higher inferred fructose metabolic activity scores in PCNSL than in either DLBCL subtype (Figure S5F). Together, these findings indicate that *SLC2A5*‐mediated fructose metabolism is specifically upregulated in PCNSL, potentially reflecting metabolic reprogramming of PCNSL tumor cells as an adaptive response to the low‐glucose environment of the CNS.

### 2.4 Characterization of T‐Cell Exhaustion in PCNSL

Next, we investigated the TME of PCNSL, initially focusing on T and NK cells. Using canonical markers, we identified four cell types, including CD4^+^ T cells, CD8^+^ T cells, NK cells, and γδ T cells across PCNSL tumor (*n* = 7), tumor‐adjacent (*n* = 3), PB (*n* = 3) and injured brain (*n* = 2) samples (Figure S6A–C). All four cell types were detected in PB samples, whereas NK and CD4^+^ T cells were nearly absent in PCNSL tumor samples (Figure S6B, D). PCNSL tumor samples were predominantly composed of CD8^+^ T cells and γδ T cells. The γδ T‐cell population, defined by the signature established by Pizzolato et al. [28], was primarily enriched in patient 6 (Figure S6E–G). A recent study showed that *B2M* defects, which impair CD8^+^ T cell‐mediated recognition of HLA class I‐bound neoepitopes, are positively associated with the infiltration of antitumor γδ T cells in DNA mismatch repair‐deficient colon cancers [29]. In line with these findings, patient 6, who exhibited the highest level of infiltrating γδ T cells, displayed marked downregulation of *B2M* expression compared to other patients (Figure S6H, I). Consistent with previous findings that Vδ2 T cells predominantly exist in PB, while Vδ2^−^ T cells are more abundant in tissues [30], we observed that Vδ2 T cells were enriched in PB, and PCNSL samples were primarily infiltrated by Vδ1 T cells (Figure S6J, K). Similar to PCNSL‐infiltrating CD8^+^ T cells, Vδ1 T cells exhibited high expression of antitumor signatures, including immune checkpoint molecules, proliferative genes and cytotoxic and effector molecules, suggesting that infiltrating γδ T cells may contribute to cytotoxic antitumor responses in PCNSL harboring HLA class I deficiencies (Figure S6L).

T cells in PCNSL have been reported to display higher exhaustion scores compared with those in systemic DLBCL [7]. To elucidate the trajectory of T‐cell exhaustion, we further classified T cells into different subtypes and performed RNA velocity analysis. Seven distinct clusters were identified and annotated as naïve T cells (Tn), tissue‐resident memory T cells (Trm), progenitor exhausted T cells (Tpex), exhausted CD8 T cells (CD8^+^ Tex1, CD8^+^ Tex2), and exhausted γδ T cells (γδ Tex1, γδ Tex2), based on their canonical marker gene expression (Figure S7A,B). There were two differentiation paths of γδ T cells, with one path progressing from naïve T cells through effector/effector memory ZNF683^+^ T cells, to Tex2 cells, and another mirroring CD8^+^ T cells from Tpex through transitional Tex1 cell state, to Tex2 cells (Figure S7A). We applied Monocle2 algorithm to reconstruct the pseudotemporal ordering of CD8^+^ T cells and γδ T cells along the shared exhaustion path (Figure S7C). As expected, the expression of Tex progenitor markers, including *TCF1* and *MKI67*, gradually declined along the exhaustion pseudotime, whereas exhaustion‐associated markers such as *PDCD1* and *HAVCR2* progressively increased (Figure S7D). Similarly, compared to transitional exhausted Tex1 cells, terminal exhausted cells Tex2 exhibited elevated expression of immune checkpoint molecules (*PDCD1*, *LAG3*, and *HACVR2*), increased exhaustion and dysfunction signature scores, and reduced proliferation signature scores (Figure S7E and Table S3) [31, 32]. Previous studies have found that glucose restriction within the TME downregulates *EZH2* expression, subsequently impairing T cell polyfunctionality and survival by limiting glycolysis [33]. Consistent with previous findings, compared to Tex1, we observed decreased *EZH2* expression and glycolysis signature scores in the more exhausted Tex2 T cell population of both γδ T and CD8^+^ T cells (Figure S7E and Table S3), suggesting that low glucose levels in PCNSL patients might play a role in T cell dysfunction. Thus, glucose restriction within the PCNSL TME not only drives metabolic reprogramming of tumor cells toward reliance on *SLC2A5*‐mediated fructose metabolism but also promotes T cell exhaustion.

### 2.5 Characterization of mo‐TAMs and mg‐TAMs in PCNSL

Myeloid cells represent another major immune cell population infiltrating PCNSL. Myeloid cells from PCNSL tumor (*n* = 7), tumor‐adjacent tissues (*n* = 3), PB (*n* = 3) and injured brain(*n* = 2) samples were classified into four subtypes, including macrophage/microglia, monocytes, neutrophils, and pDCs, based on canonical cell‐type markers (Figure S8A–C). Myeloid cells in PB predominantly consisted of monocytes (99.4%) (Figure S8D). In injured brain tissues, myeloid cells comprised both macrophage/microglia (59.0%) and infiltrating monocytes (29.7%) (Figure S8D). Macrophages/microglia constituted the dominant myeloid cell population in PCNSL and tumor‐adjacent tissues, accounting for 97.14% and 91.10% of total myeloid cells, respectively (Figure S8D).

We next characterized macrophages/microglia in PCNSL tumor and tumor adjacent tissues. These cells were further subdivided into microglia‐derived tumor‐associated macrophages (mg‐TAMs) /microglia and monocyte‐derived tumor‐associated macrophages (mo‐TAMs) based on their expression profiles (Figure 4A) [34, 35, 36]. A single‐cell transcriptomic analysis of 51 diffuse glioma cases identified *P2RY12*, *CX3CR1*, *FCGR1A*, *CH25H*, and *CCL4L2* as mg‐TAM‐specific markers, whereas *TGFBI*, *CD14*, *CD163*, *SELENOP*, and *GPNMB* were designated as mo‐TAM‐specific markers [36]. A separate study comparing the single‐cell transcriptomic profiles of mg‐TAMs and mo‐TAMs in newly diagnosed glioblastoma patients reported *TMIGD3*, *RNASET2*, *BIN1*, *TREM2*, *CX3CR1*, *TMEM119*, *CALM2*, *SYNDIG1*, *FSCN1*, *SPP1*, *DHRS9*, *SCIN*, *APBB1IP*, *FCGR1A*, and *BHLHE41* as the 15 most significantly upregulated genes in mg‐TAMs, whereas *VIM*, *S100A10*, *TGFBI*, *LYZ*, *FTH1*, *ANXA1*, *LGALS3*, *S100A6*, *CD44*, *CTSD*, *LGALS1*, *NAMPT*, *S100A4*, *CST3*, and *SAT1* were the 15 most significantly upregulated genes in mo‐TAMs [34]. In recurrent glioblastoma tumors from the same study, *TMEM119*, *P2RY12*, *SALL1*, *TMIGD3*, *APOC2*, and *SCIN* were also among the top upregulated genes in mg‐TAMs, while *TGFBI, CLEC12A*, and *FXYD5* were prominently upregulated in mo‐TAMs [34]. In our PCNSL cohort, mg‐TAMs/microglia and mo‐TAMs expressed canonical macrophage markers, including *C1QA*, *C1QB*, and *C1QC* (Figure 4B). Consistent with the transcriptomic signatures observed in glioma and glioblastoma, mg‐TAMs/microglia in PCNSL specifically expressed canonical microglial signature genes, such as *TMEM119* and *P2RY12* (Figure 4B–D), supporting a microglial ontogeny. In contrast, mo‐TAMs expressed genes previously reported to be associated with monocyte‐derived brain macrophages (e.g., *TGFBI* and *CLEC12A*) (Figure 4B–D) [34]. A direct comparison of mo‐TAMs and mg‐TAMs within PCNSL samples further confirmed this distinction, with mg‐TAMs preferentially expressing microglia‐related genes and mo‐TAMs expressing monocyte‐associated genes (Figure S8E). Differential gene expression patterns between mo‐TAMs and mg‐TAMs in PCNSL are consistent with observations in glioblastoma. Genes previously reported to be enriched in mg‐TAMs relative to mo‐TAMs, such as *TREM2*, *SCIN*, *TMIGD3*, *SPP1*, and *CH25H*, also exhibited higher expression in PCNSL mg‐TAMs (Figure 4C; Figure S8E). Conversely, genes associated with mo‐TAMs in glioblastoma, including *CD14*, *CD163*, *CD44*, *LYZ*, *ANXA1*, and *CLEC12A*, were preferentially expressed in mo‐TAMs in PCNSL (Figure 4B,C; Figure S8E) [34, 36].

**FIGURE 4:**
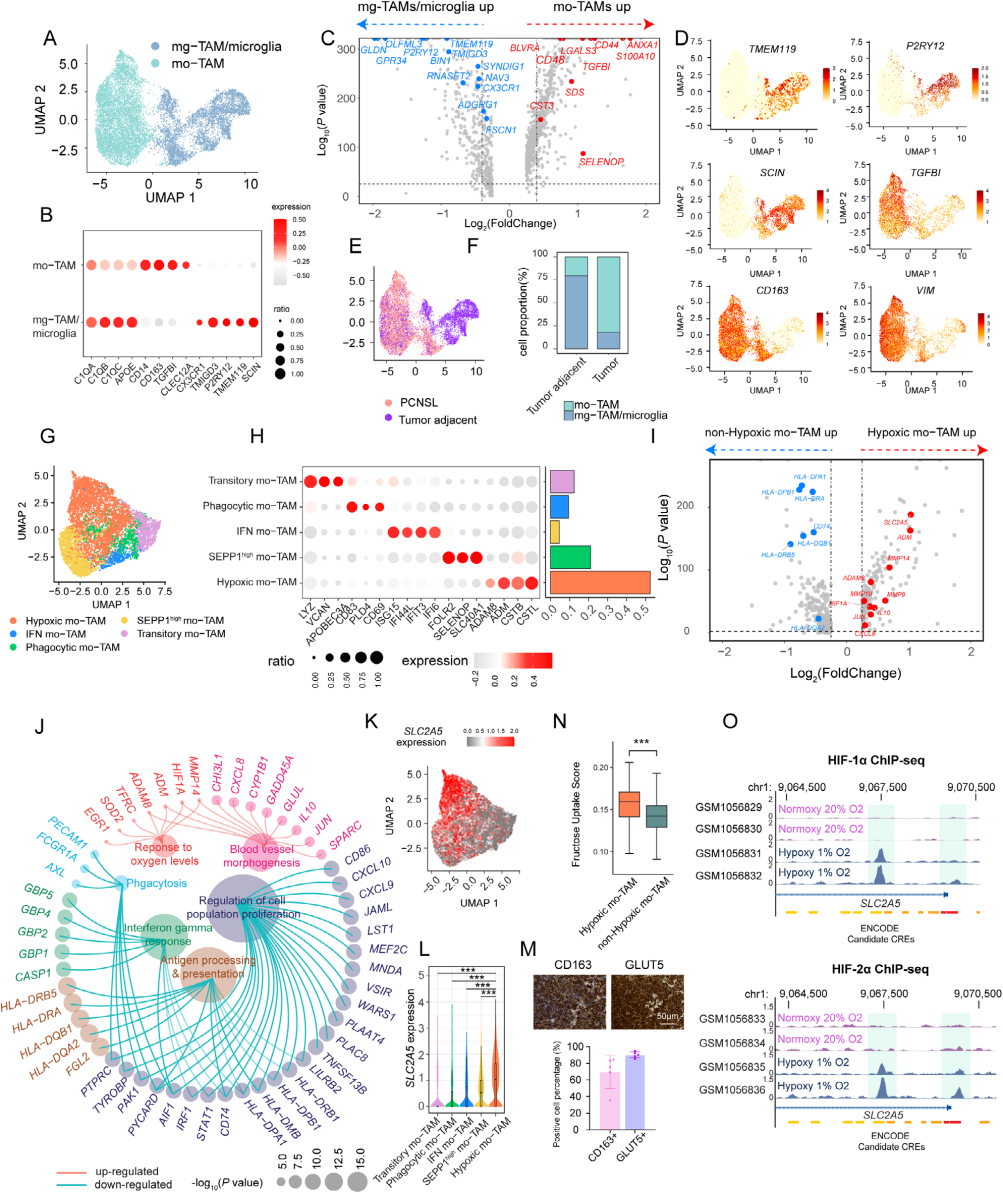
Characterization of macrophage/microglia cells in PCNSL. (A) UMAP visualization of microglia and tumor associated macrophages derived from PCNSL tumor samples from 7 patients and tumor‐adjacent samples from 3 patients. Each dot represents a single cell, colored by cell types. (B) Expression of representative marker genes of mo‐TAMs and mg‐TAMs/microglia. (C) Volcano plot showing differentially expressed genes between mo‐TAMs and mg‐TAMs/microglia. (D) UMAP visualization of representive mo‐TAM and mg‐TAM/microglia marker gene expression levels. (E) UMAP visualization of tumor associated macrophages/microglia. Each dot represents a single cell, colored by sample origins. (F) Proportions of mo‐TAMs and mg‐TAMs/microglia among total TAMs in tumor and tumor‐adjacent samples. (G) UMAP visualization of mo‐TAMs from PCNSL tumor samples from 7 patients, colored by distinct mo‐TAM subtypes. (H) Expression of representative marker genes across mo‐TAM subtypes. The bar plot (right) shows the proportion of each mo‐TAM subtype among total mo‐TAMs in PCNSL samples. (I) Volcano plot showing differentially expressed genes between hypoxic mo‐TAMs and non‐ hypoxic mo‐TAMs. (J) Circle plot showing significantly enriched pathways in hypoxic mo‐TAMs compared to non‐ hypoxic mo‐TAMs. Red lines indicate upregulated pathways, and blue lines indicate downregulated pathways. Circle size indicates statistical significance of each enriched pathway. (K) UMAP visualization of *SLC2A5* expression levels in mo‐TAMs. (L) *SLC2A5* expression levels across mo‐TAM subtypes. Statistical significance was assessed using two‐sided Student's t‐tests (***p < 0.001). (M) IHC staining of CD163 and GLUT5 showing that regions with high CD163 expression exhibit strong GLUT5 expression. Quantification of the percentages of CD163‐positive and GLUT5‐positive cells is shown below in the bar plot. Each dot represents an individual sample (*n* = 5), and bars indicate mean ± SD. Differences in the percentages of CD163‐positive cells and GLUT5‐positive cells across the five samples were accessed using Wilcoxon test (*p* = 0.008) (N) Inferred fructose uptake scores in hypoxic and non‐hypoxic mo‐TAMs calculated using METAFlux. Differences between two groups were evaluated using by the Wilcoxon test, with *** indicating p < 0.001. (O) Density maps are shown for HIF‐1α and HIF‐2α ChIP‐seq at *SLC2A5* locus (hg38) in primary human blood‐derived macrophages cultured under normoxic (20% O2) or hypoxic (1% O2) conditions. ENCODE‐predicted enhancers and promoters are indicated by orange and red lines, respectively.

Although both microglia/mg‐TAMs from PCNSL and tumor‐adjacent tissues retain a microglial ontogeny, PCNSL mg‐TAMs exhibited downregulation of genes, including *PYRY12*, *TMEM119*, and *CX3CR1* and upregulation of genes, such as *CD163* and *IFI27* (Figure S8F), compared with tumor‐adjacent tissues. This indicates that PCNSL mg‐TAMs display reduced expression of homeostatic microglial genes and a concomitant increase in inflammatory gene programs relative to microglia in tumor‐adjacent tissue (Figure S8G). These observations are consistent with previous findings in glioma‐associated microglia relative to microglia from non‐tumor brain tissues [37].

Regarding the spatial localization of TAM subsets, mo‐TAMs were predominantly enriched in PCNSL tumor samples (81.8%), whereas mg‐TAMs/microglia (79.3%) were mainly localized to tumor‐adjacent tissues (Figure 4E,F). This distribution mirrors the spatial organization observed in glioblastoma, where mg‐TAMs are enriched at the invasive tumor margin, while mo‐TAMs are primarily confined to the tumor core [34, 38, 39]. Collectively, mo‐TAMs and mg‐TAMs/microglia exhibit distinct ontogenetic features and spatial localization preferences in PCNSL.

### 2.6 Hypoxic mo‐TAMs Enriched in the Microenvironment of PCNSL Show Upregulation of Fructose Transporter Gene *SLC2A5*

Mo‐TAMs represent the predominant myeloid population in PCNSL tumor samples (Figure 4F). We subsequently examined the molecular and functional heterogeneity of mo‐TAMs within PCNSL microenvironment. Five transcriptionally distinct mo‐TAM clusters, transitory, phagocytic, IFN, SEPP1^high^, and hypoxic mo‐TAMs, were identified based on their marker gene expression profiles (Figure 4G, H) [34, 36, 40]. Hypoxic mo‐TAMs constituted the largest proportion of mo‐TAMs in PCNSL. Compared to non‐hypoxic mo‐TAMs, hypoxic mo‐TAMs showed upregulation of hypoxia‐related genes (*HIF1A*, *JUN*, *ADM*, *ADAM8*), matrix metalloproteinases (*MMP9*), angiogenic factors (*CXCL8*), and cytokines (*IL10*) (Figure 4I). MMPs released by hypoxic macrophages have been shown to enhance tumor cell migration and invasion in breast cancer [41]. *IL‐10* has been reported to induce M2‐like macrophage functions, including anti‐inflammation, pro‐tumorigenesis, and pro‐angiogenesis [42]. In agreement with previous studies indicating that TAMs with low MHC‐II expression are enriched in hypoxic regions, we observed downregulation of MHC‐II molecules (e.g., *HLA‐DPA1*, *HLA‐DPB1*, and *HLA‐DRA*) in hypoxic mo‐TAMs (Figure 4I) [43]. Pathway enrichment analysis further indicated the tumor‐supportive roles of hypoxic mo‐TAMs in PCNSL. Hypoxic mo‐TAMs exhibited significant downregulation of pathways related to phagocytosis, interferon‐gamma response, cell population proliferation, and antigen processing and presentation, while pathways associated with oxygen level response and blood vessel morphogenesis were markedly upregulated (Figure 4J). This aligns with previous findings in glioblastoma, where hypoxic niches feature abundant but structurally destabilized blood vessels, and hypoxia‐TAMs promote hyperpermeable vasculature that impedes effective drug delivery [36].

Interestingly, we observed that the *SLC2A5* gene was also significantly upregulated in hypoxic mo‐TAMs (Figure 4I,K,L). We further examined *SLC2A5* expression at the protein level, and IHC staining of clinical PCNSL tissue sections using antibodies against CD163 and GLUT5 revealed that regions with high CD163 expression also exhibited strong GLUT5 staining (Figure 4M; Figure S5A). Furthermore, the METAFlux‐inferred fructose uptake score was also higher in hypoxic mo‐TAMs compared with non‐hypoxic mo‐TAMs (Figure 4N). To further explore whether hypoxic conditions within the TME drive fructose utilization in mo‐TAMs, we analyzed public ChIP‐seq data of hypoxia‐inducible factors HIF‐1α and HIF‐2α in primary human blood‐derived macrophages cultured under different oxygen concentrations [44, 45]. The data demonstrated that HIF‐1α and HIF‐2α bind to the promoter and/or putative enhancer regions of *SLC2A5* under hypoxic conditions, suggesting that hypoxia within the TME may induce *SLC2A5* expression through HIF‐mediated transcriptional regulation (Figure 4O). These findings highlight GLUT5, encoded by *SLC2A5*, and its associated fructose metabolic pathways as potential therapeutic targets, not only for tumor cells but also for tumor‐supportive hypoxic mo‐TAMs within the TME.

### 2.7 Blockage of Fructose Utilization Impairs B‐Cell Lymphoma Cells under Low‐Glucose Conditions

To further evaluate whether GLUT5 represents a metabolic vulnerability in PCNSL, we conducted both in vivo and in vitro experiments. We first assessed whether fructose uptake promotes B‐cell lymphoma proliferation under glucose‐deficient conditions by culturing the DLBCL cell line U‐2932 under varying glucose concentrations. The results demonstrated that fructose supplementation significantly promoted cell proliferation under low‐glucose or glucose‐deprived conditions, whereas the effect was minimal or absent under normal or high‐glucose conditions (Figure 5A).

**FIGURE 5:**
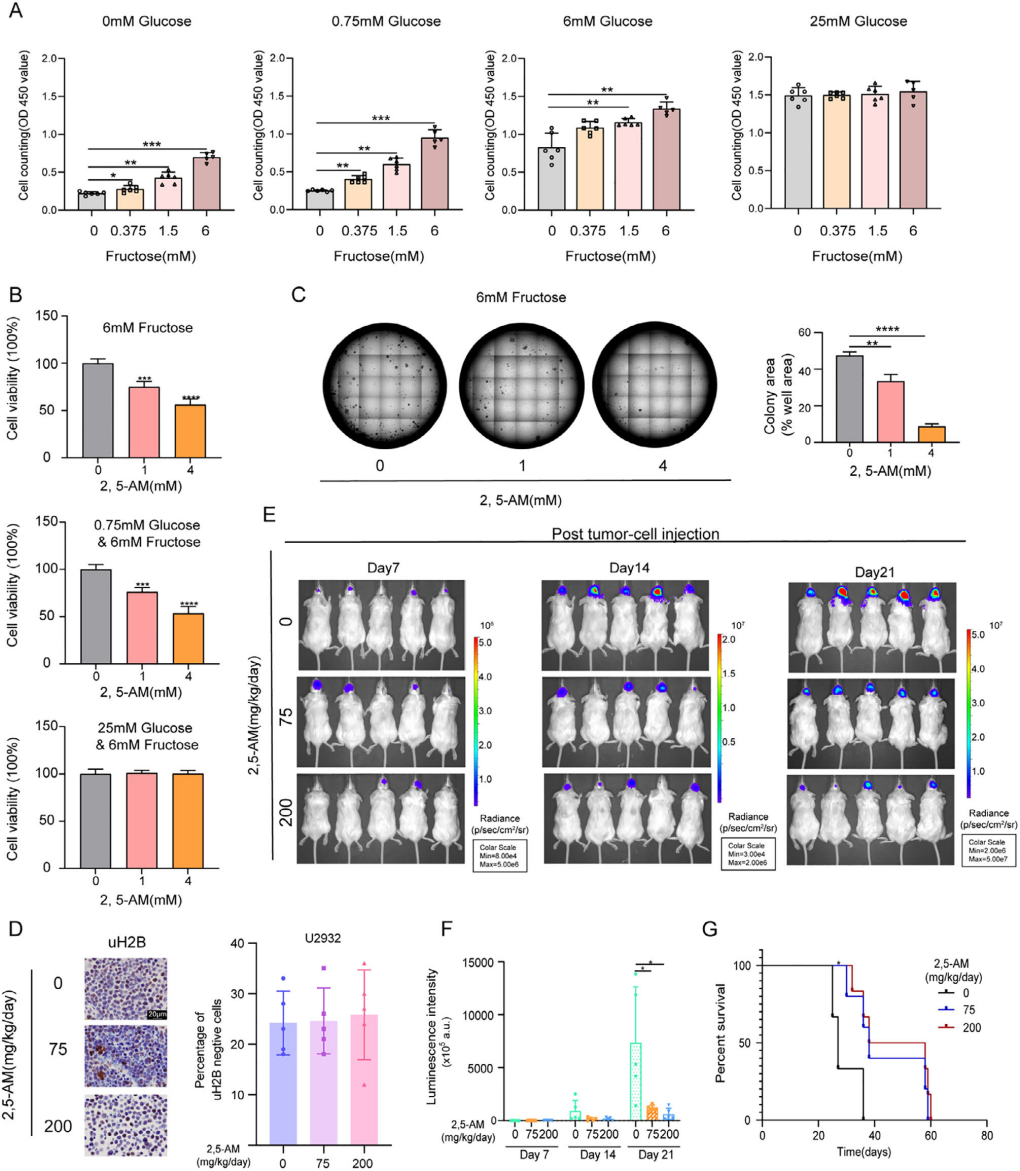
Pharmacological inhibition of fructose utilization suppresses lymphoma growth under low‐glucose conditions. (A) Fructose‐induced proliferation of U‐2932 cells under different glucose concentrations. Cells were cultured in the indicated media for 48 h. For each glucose condition, cell proliferation at different fructose concentrations (0.375 mm, 1.5 mm, and 6 mm) was compared to that at 0 mm fructose. Statistical significance was assessed using Student's t‐test, with * indicating *p* < 0.05, ** indicating *p* < 0.01, and *** indicating *p* < 0.001. (B) U‐2932 cells cultured in the indicated media were treated with different concentrations of 2,5‐AM (0, 1, and 4 mm). Cells were harvested and counted after 3 d. (C) U‐2932 cells cultured in glucose‐deprived medium supplemented with 6 mm fructose were treated with 2,5‐AM of different concentrations (0, 1, and 4 mm). Cells were harvested and assessed for colony formation in MethoCult H4230 medium after 14 d. (D) Representative IHC images of uH2B staining (left). Glucose deficiency was assessed by calculating the percentage of uH2B‐negative cells across 40 microscopic fields per tumor sample (right). Data represent mean ± SEM for each group (*n* = 5). (E) Luciferase‐expressing U‐2932 cells were intracranially injected into immunodeficient mice (*n* = 5 per group). Seven days after tumor‐cell injection, mice were treated daily with 200 µL of 2,5‐ AM (75 or 200 mg kg^−1^) or vehicle (PBS) via intraperitoneal administration for 14 d. Tumor‐ derived luminescence intensity was measured at the indicated time points after tumor‐cell injection, and relative luminescence intensity is shown. a.u., arbitrary units. (F) Bar plot summarizing relative luminescence intensity at different time points post tumor‐cell injection in mice treated with vehicle or different doses of 2,5‐AM. Data represent mean ± SEM for each group (*n* = 5), with comparisons made between treatment and vehicle groups at each time point. Asterisks indicate statistical significance (*p < 0.05, **p < 0.01, ***p < 0.001). (G) Luciferase‐expressing U‐2932 cells were intracranially injected into immunodeficient mice (*n* = 10). Seven days after tumor‐cell injection, 200 µL of 2,5‐AM (75 or 200 mg kg^−1^) or vehicle (PBS) was delivered to the mice via intraperitoneal administration daily. The survival times of the mice were recorded.

Next we investigated whether inhibiting fructose utilization could impair B‐cell lymphoma cell growth under glucose‐deficient conditions. We first generated *SLC2A5*‐knockout U‐2932 cells and cultured them under different glucose concentrations (Figure S9A). The results demonstrated that the enhanced cell proliferation and colony formation observed upon fructose supplementation under glucose‐deprived conditions was abolished by *SLC2A5* knockout (Figure S9B,C). To access whether *SLC2A5* knockout impairs fructose uptake, we performed fructose uptake assays in control and *SLC2A5*‐knockout U‐2932 cells. We found that *SLC2A5* knockout led to reduced fructose consumption compared with wild‐type (WT) cells under glucose‐deficient condition (Figure S9D), indicating that fructose‐induced lymphoma growth under glucose‐limited conditions is facilitated by *SLC2A5*‐mediated fructose uptake. Furthermore, we treated U‐2932 cells with 2,5‐anhydro‐D‐mannitol (2,5‐AM), a fructose analog with high affinity for GLUT5 that inhibits fructose uptake, under varying glucose concentrations. 2,5‐AM treatment also significantly inhibited fructose‐stimulated cell proliferation and colony formation under glucose‐deficient conditions (Figure 5B,C).

To determine whether blockage of fructose utilization suppresses PCNSL growth in vivo, we employed an orthotopic model of PCNSL [46, 47]. Specifically, luciferase‐expressing U‐2932 cells were intracranially injected into NOD‐*Prkdc^scid^IL2rg^em9^* (NSIG) mice, which were subsequently treated with PBS or 2,5‐AM at low or high doses. We first assessed glucose levels in tumor specimens by examining uH2B (K120 mono‐ubiquitination of histone H2B), a semiquantitative histone marker reflecting glucose deprivation in tumor tissues [48]. IHC staining of uH2B in tumor sections from each treatment group revealed that approximately 20%–40% of U‐2932 tumor cells were negative for uH2B, indicating that a substantial proportion of tumor cells were experiencing glucose deprivation (Figure 5D). Bioluminescence imaging of tumor‐bearing mice treated with 2,5‐AM revealed a marked reduction in lymphoma cell growth within the brain (Figure 5E,F). Additionally, *SLC2A5*‐knockout luciferase‐expressing U‐2932 cells were generated and injected into NSIG mice. Bioluminescence imaging demonstrated that *SLC2A5* knockout also suppressed lymphoma cell growth significantly (Figure S9E,F). Both tumor‐bearing mice treated with 2,5‐AM and mice injected with *SLC2A5*‐knockout cells showed significantly prolonged overall survival compared to control groups (Figure 5G; Figure S9G).

Together, these findings indicate that genetic or pharmacologic inhibition of fructose utilization suppresses lymphoma growth under low‐glucose conditions both in vitro and in vivo, highlighting fructose metabolism as a potential therapeutic vulnerability in PCNSL.

## 3 Discussion

Fructose consumption has increased substantially over the past five decades, largely due to its incorporation into processed foods in the form of sucrose or high‐fructose corn syrup [49]. Cancer cells can utilize fructose as an alternative energy source, with many tumor types enhancing fructose uptake through upregulation of the fructose‐specific transporter GLUT5. For example, a positive association has been reported between fructose intake and the risk of pancreatic cancer [50]. In addition, elevated GLUT5 expression has been observed in lung adenocarcinoma, acute myeloid leukemia, and glioma, where higher GLUT5 levels correlate with poor prognosis [51, 52, 53]. CNS lymphoma is recognized as a disease associated with low CSF glucose levels [9, 10]; however, the extent to which glucose deficiency within the CNS TME drives metabolic rewiring in cancer cells remains largely unexplored. Here, using single‐cell multi‐omic approaches, we comprehensively characterized tumor and non‐malignant microenvironment cell populations in PCNSL. Our analyses revealed, for the first time, the upregulation of the fructose transporter *SLC2A5* in PCNSL. By applying METAFlux, a computational framework that infers metabolic fluxes from single‐cell transcriptomic data, we showed increased METAFlux‐inferred fructose‐associated metabolic activity in PCNSL, including the conversion from fructose to fructose 1‐phosphate or fructose 6‐phosphate, as well as the conversion of glyceraldehyde to GA3P. These findings indicated that glucose deprivation reprograms PCNSL cells to depend on GLUT5‐mediated fructose metabolism as an alternative energy source. However, how fructose is incorporated into glycolytic intermediates and other metabolic pathways to support PCNSL tumor cell growth remains to be elucidated in future studies. Recent studies suggested that, in addition to GLUT5‐mediated fructose uptake, endogenous fructose production from glucose via the AKR1B1‐mediated polyol pathway represents an alternative route for fructose utilization in pancreatic cancer cells [54, 55]. Whether PCNSL also engages the polyol pathway to sustain fructose metabolism and promote tumor growth also remains to be determined.

Most studies have focused on the direct effects of fructose metabolism on tumor cells. Although immune cells within the TME also play critical roles in tumor progression, the impact of fructose metabolism on the TME cells remains poorly understood. In our study, we observed that hypoxic mo‐TAMs were specifically enriched in PCNSL and exhibited tumor‐supportive signatures, characterized by downregulation of pathways involved in phagocytosis, interferon‐gamma response, cell proliferation, and antigen processing and presentation, alongside upregulation of pathways related to oxygen response and blood vessel morphogenesis. Notably, *SLC2A5* was significantly upregulated in hypoxic mo‐TAMs, and we further demonstrated that hypoxia induces *SLC2A5* expression in macrophages through HIF‐mediated transcriptional regulation. A recent study found that fructose promotes colorectal cancer tumorigenesis by inhibiting M1‐like macrophage polarization. Fructose acts as a signaling molecule to enhance the interaction between hexokinase 2 (HK2) and inositol 1,4,5‐trisphosphate receptor type 3 (ITPR3), the primary Ca^2^^+^ channel of the endoplasmic reticulum. This interaction decreases Ca^2^^+^ levels in the cytosol and mitochondria, consequently suppressing the activation of MAPK, STAT1, and the NLRP3 inflammasome, ultimately impeding the polarization of M1‐like macrophage [56]. Our study also suggested that GLUT5‐mediated fructose metabolism may represent a potential metabolic vulnerability in tumor‐associated macrophages within the PCNSL TME. However, future studies involving protein‐level and functional validation of *SLC2A5* in hypoxic mo‐TAMs from PCNSL specimens, together with in vitro and in vivo studies, will be required to further evaluate the therapeutic potential of targeting GLUT5‐mediated fructose metabolism in these macrophages. In addition, the mechanisms by which fructose metabolism promotes the tumor‐supportive functions of hypoxic mo‐TAMs remain to be elucidated.

Taken together, our study described fructose metabolism as a potential metabolic vulnerability, targeting not only tumor cells but also tumor‐supportive hypoxic mo‐TAMs within the TME. In vitro and in vivo experiments demonstrated that inhibition of fructose uptake markedly suppressed lymphoma growth. Although these findings were based on a relatively small discovery cohort, we provide preliminary evidence that pharmacological inhibition of fructose metabolism or dietary fructose restriction may represent promising therapeutic strategies for patients with PCNSL. Future studies incorporating larger and more diverse patient cohorts will be important to validate these observations and to evaluate their broader applicability to PCNSL.

## 4 Methods

### 4.1 Clinical Samples and Sequencing Strategy

Clinical samples were collected from seven patients diagnosed with PCNSL and two individuals with traumatic brain injuries (Table S1). Magnetic resonance imaging revealed solitary lesions in all PCNSL cases. scRNA‐seq was performed on fresh samples obtained from surgical resections or stereotactic biopsies derived from 4 PCNSL patients (3 with paired tumor and tumor‐adjacent samples and 1 with tumor sample only) and 2 patients with traumatic brain injuries. scRNA‐seq coupled with scBCR‐seq was conducted on tumor samples and matched PB samples from another 3 PCNSL cases. Informed consent was obtained from all participants prior to sample collection.

### 4.2 Tissue Dissociation and Single‐Cell Suspension Preparation

Fresh samples obtained via needle biopsy or surgical resections were immediately preserved in the GEXSCOPE tissue preservation solution (Singleron) at 2–8 °C. Hanks balanced salt solution (HBSS) was used to wash the specimens for three times. The samples were then minced into 1–2 mm pieces, and subsequently subject to enzymatic digestion using 2 mL of GEXSCOPE tissue dissociation solution (Singleron) at 37°C for 15 min in a 15 mL centrifuge tube with continuous agitation. After digestion, the samples were filtered through 40 µm sterile strainers and centrifuged at 1000 rpm for 5 min. The supernatant was then discarded, and 1 mL of PBS (HyClone) was used to resuspend cell pellets.

### 4.3 ScRNA‐Seq Library Preparation

Single‐cell suspensions at a concentration of 1×10^5^ cells mL^−1^ were prepared in PBS (HyClone). These suspensions were subsequently loaded onto microfluidic chip, and scRNA‐seq libraries were constructed following the Singleron GEXSCOPE protocol using the GEXSCOPE single‐cell RNA Library Kit (Singleron Biotechnologies) [57]. Individual libraries were diluted to a final concentration of 4 nm and pooled for sequencing. The pooled libraries were sequenced on an Illumina NovaSeq 6000 platform using 150 bp paired‐end reads.

### 4.4 scBCR‐Seq Library Preparation

The single‐cell suspension (1×10^5^ cells mL^−1^) was loaded onto microfluidic devices, and scBCR‐seq libraries were constructed following the manufacturer's protocol of the GEXSCOPE single cell human V(D)J Kit (Singleron Biotechnologies). In brief, following cell lysis, magnetic beads conjugated with molecular barcodes captured the poly(A) tails and BCR regions of mRNA, enabling the labeling of individual cells and individual mRNA molecules. Afterwards, the magnetic beads within the chip were collected, and the mRNA captured by the beads was reverse transcribed into complementary DNA (cDNA) and subsequently amplified. A portion of the cDNA was utilized to construct sequencing libraries compatible with the Illumina platform. The remaining cDNA was subjected to BCR enrichment, and the enriched products were subsequently amplified by PCR to generate sequencing libraries for the Illumina platform. Finally, each library was sequenced on the NovaSeq 6000 platform using 150 bp paired‐end reads.

### 4.5 Expression Matrix Generation

Raw sequencing reads were processed using CeleScope tool to generate gene expression matrix (Https://Github.com/Singleron‐RD/CeleScope). Briefly, read one sequences lacking poly‐T tails were first filtered out, and the cell barcodes and unique molecular identifiers (UMIs) were extracted. Adapters and poly‐A tails were trimmed, followed by alignment of read two to the GRCh38 reference genome with Ensembl version 99 gene annotation. Reads sharing identical cell barcodes and gene annotations were grouped to quantify the number of UMIs per gene per cell. The number of cells was subsequently determined using the EmptyDrops method [58]. The resulting UMI count matrices for each cellular barcode and each gene were used for downstream analyses.

### 4.6 ScRNA‐Seq Data Processing

Single‐cell data analysis was performed using the Seurat package (version 4.4.0) [59]. Genes detected in at least three cells were kept for further analysis. Low‐quality cells were filtered based on the following criteria: (1) cells with fewer than 200 or more than 7000 detected genes were excluded; (2) cells with mitochondrial gene expression exceeding 10% to 30% were removed, with thresholds determined based on the mitochondrial gene percentage distribution of each sample; and (3) potential doublets, identified as cells co‐expressing canonical markers of multiple distinct cell types, were manually excluded. To eliminate ambient RNA contamination, the expression matrix was further corrected using the DecontX package (version 1.0.0) [60]. After quality control, a total of 140844 single cells across all samples were retained for downstream analyses.

### 4.7 Clustering and Annotation

The Seurat package (version 4.4.0) [59] was utilized for single‐cell data normalization, dimensionality reduction, and unsupervised clustering. In brief, 2000 highly variable genes were identified and used for downstream analyses. Principal component analysis (PCA) was conducted using the RunPCA function. A k‐nearest neighbor (KNN) graph was constructed based on the first 50 principal components (PCs) to perform clustering analysis, implemented using the FindNeighbors and FindClusters functions with the resolution parameter set to 0.5. UMAP was employed for visualization of the single‐cell RNA‐seq data.

Cell types were annotated based on the expression of following canonical marker genes: B cells (*MS4A1*, *CD79A*, *CD79B*), plasmablasts (*SDC1*, *IGHG1*, *TNFRSF17*), T cells (*CD3E*, *CD3D*, *CD3G*), NK cells (*FCGR3A*, *KLRB1*, *KLRF1*), monocytes(*LYZ*, *VCAN*, *FCN1*) macrophage/microglia (*C1QA*, *C1QB*, *C1QC*), neutrophils (*CSF3R*, *FCGR3B*,*S100A8*), pDCs (*IRF7*, *PTPRS*, *LILRA4*), mural cells (*NDUFA4L2*, *PDGFRB*, *ACTA2*), endothelial cells (*CDH5*, *CAVIN2*, *CLDN5*), oligodendrocytes (*MAG*, *MAL*, *ERMN*), oligodendrocyte progenitor cells (OPCs) (*PDGFRA*, *GPR17*, *TNR*) and astrocytes (*ALDH1L1*, *ETNPPL*, *CHI3L1*).

A second round of clustering was performed on B cells, T cells, and myeloid cells (including monocytes, macrophages, and pDCs) from PCNSL tumor samples to investigate inter‐patient heterogeneity of each cell type. For reclustering, the top 20 PCs were used, with the resolution parameter set to 0.1 for B and myeloid cells, and 0.3 for T cells.

### 4.8 scRNA‐Seq‐Based CNV Identification

CNVs in B cells from tumor and tumor‐adjacent samples were inferred using inferCNV (version 1.16.0) [61]. CNV analysis was performed separately for each patient with the following parameters: analysis_module = ‘subclusters’, cutoff = 0.1, and tumor_subcluster_partition_method = ‘random_trees’. T cells from the same individual were used as a normal reference to estimate CNVs in B cells. For representative CNV visualization, heatmaps displaying relative expression intensities across chromosomes were generated using all B cells from patients 1 and 2, and 50% of B cells randomly sampled from patient 3. Phylogenetic trees illustrating the evolutionary trajectories of tumor B cells were constructed using the Python package UPhyloplot2 (version 2.2.3) [62].

### 4.9 Annotation of B Cell Subtypes via Mapping to External scRNA‐Seq Reference

A B cell reference was constructed using published scRNA‐seq data from normal tonsils, which comprises 12 annotated B cell subtypes [12]. The FindTransferAnchors and MapQuery functions from the Seurat package were applied to transfer B cell subtype annotations from the reference to our dataset and to project B cells from our samples onto the UMAP structure of the reference.

### 4.10 Identification of Recurrent Expression Programs

An NMF‐based approach was employed to extract expression programs of malignant B cells in each patient using the R package GeneNMF (version 0.4.0) [63]. NMF programs were identified using the multiNMF function with parameters *K = 5* and nfeatures *=* 2000. NMF programs derived from all patients were subsequently clustered to identify meta‐programs using the getMetaPrograms function with the parameter nMP = 10. Meta‐programs detected in at least three patients were visualized via heatmap. The runGSEA function was utilized to identify Gene Ontology (GO) pathways significantly enriched in each meta‐program. The AddModuleScore_UCell function from the Seurat package was employed to calculate meta‐program scores for each cell, using the most influential genes identified by GeneNMF for each meta‐program.

### 4.11 ScBCR‐Seq Data Analysis

The scRepertoire (v1.10.1) package was utilized for scBCR‐seq data analysis [64]. Cells expressing both heavy and light BCR chains were retained for downstream analysis. Clonal categorization was based on the amino acid sequences of the BCRs. Clonotypes were defined according to the number of identical amino acid sequences as follows: Hyperexpanded (*X* > 100), large (20 < *X* ≤ 100), medium (5 < *X* ≤ 20), small (1 < *X* ≤ 5), and single (0< X ≤ 1). Detailed information on BCR chains (amino acid sequences, gene usage, and nucleotide sequences) and the distribution of clonotypes is provided in Table S2.

### 4.12 Annotation of Γδ T Cells and Their Subtypes

A gene signature developed by Pizzolato et al. was employed to score individual cells and annotate γδ T cells [28]. Specifically, the γδ signature score was derived from the combinatorial expression of two gene sets: GS1 = (*CD3D*, *CD3E*, *TRDC*, *TRGC1*, *TRGC2*) and GS2 = (*CD8A*, *CD8B*). The GS1 score for each cell was calculated as: (the sum of raw UMI counts for all GS1 genes expressed in the cell) / (the total UMI count of that cell). The GS2 score for an individual cell was computed as: ‐(the sum of raw UMI counts for GS2 genes expressed in the cell) / (the total UMI count of that cell). The normalized GS1 score for a cell was calculated as: (GS1 score of that cell—minimum GS1 score across all cells) / (maximum GS1 score across all cells—minimum GS1 score across all cells). The normalized GS2 score was calculated in the same manner: (GS2 score of that cell—minimum GS2 score across all cells) / (maximum GS2 score across all cells—minimum GS2 score across all cells). The γδ signature score for each cell was defined as the product of its normalized GS1 and GS2 scores. Cells with a γδ signature score exceeding 0.35 were annotated as γδ T cells.

γδ T cell subtypes were annotated based on the expression levels of *TRGC1* and *TRGC2*, calculated using Seurat‐normalized UMI counts without log2 transformation. Among the γδ T cell population, cells with *TRGC1*–*TRGC2* < 0 were classified as Vδ2, those with *TRGC1 – TRGC2* > 0 as Vδ1, and cells with *TRGC1* – *TRGC2* = 0 were labeled as undetermined.

### 4.13 Infer Metabolic Fluxes from Single‐Cell Transcriptomic Data

METAFlux [26], an R package that leverages a genome‐scale metabolic model to capture the mechanistic relationships among genes, metabolites, and biochemical reactions in human cells, was employed to infer metabolic fluxes of B cells from PCNSL, DLBCL, and PB samples based on scRNA‐seq data. Fluxes scores of metabolite uptake/release and metabolic reactions involved in fructose and glucose metabolism were compared across B cells from PCNSL, DLBCL, and PB samples. Specifically, the irreversible reactions with the following HMR models *IDs—*HMR_9139, HMR_4310, HMR_4319, HMR_4379, HMR_0454—were analyzed. All reaction IDs were subsequently converted to human‐GEM IDs.

### 4.14 RNA Velocity and Pseudotime Analyses

RNA velocity analysis was performed using the Python packages velocyto and scVelo (version 0.2.5) [65, 66]. Velocyto was used to quantify spliced and unspliced transcript counts from aligned BAM files, and RNA velocities were subsequently estimated and embedded into UMAP space as streamlines using scVelo.

Pseudotime analysis was conducted using the Monocle2 R package (version 2.21.1) [67]. The reduceDimension function was used to project the data into two dimensions with the parameters max_components = 2 and method = ‘DDRTree’. Cell trajectories were inferred using the orderCells function. Pseudotime trajectories and gene expression dynamics were visualized using the plot_cell_trajectory and plot_genes_in_pseudotime functions.

### 4.15 Cells and Cell Culture

The human B cell lymphoma cell line U‐2932 was cultured in RPMI 1640 medium (Gibco, Thermo Fisher Scientific, USA) supplemented with 10% heat‐inactivated fetal bovine serum (FBS; OriCell, Cyagen Biosciences, China) and 1% penicillin/streptomycin solution (Invitrogen, Thermo Fisher Scientific, USA). Cells were cultured at 37°C in a humidified incubator with 5% CO_2_ and 95% air. The cell line used in this study was not found in the database of commonly misidentified cell lines maintained by the International Cell Line Authentication Committee and the National Center for Biotechnology Information BioSample. The cell line is tested negative for mycoplasma contamination.

### 4.16 Stable Knockout Cell Line Generation

For *SLC2A5* KO, single guide RNAs (sgRNAs) were designed using the online CRISPR design tool (Red Cotton, Guangzhou, China, https://en.rc‐crispr.com/). The region within exon 5 of *SLC2A5* was selected as the target site for CRISPR/Cas9‐mediated genome editing. Cas9 protein and sgRNA were incubated at room temperature for 10–20 min, followed by electrotransfection into a total of 3 × 10^5^ U‐2932 cells according to the manufacturer's instructions. 48 h after electroporation, cells were subjected to limiting dilution and inoculated into 96‐well plates. Individual clones were screened 2–4 weeks post‐plating, and knockout clones were validated by PCR and Sanger sequencing. The sgRNA sequences targeting *SLC2A5* are as follows:

*SLC2A5*‐SgRNA‐1: GCCAGCTCCCCTAAGTACAT
*SLC2A5*‐SgRNA‐2: CCCCAGCTCTTCATCACTGT

The primers used for PCR validation are as follows:

Forward Primer (F1): GAAACGTGAGTGGGTCTAGAC
Reverse Primer 1 (R1): GAGGGACTAGATAGTAAACGCTG
Reverse Primer 2 (R2): GTGATGAAGAGCTGGGGCAC

### 4.17 RNA Isolation and Quantitative PCR

One microgram of total RNA, extracted with TRIzol Reagent (Cat. #19201ES; Yeasen Biotechnology, Shanghai, China), was reverse‐transcribed in a 20 µL reaction containing 4 µL of 5× gDNA Digestion Mix (Cat. #11141ES; Yeasen) and 5 µL of 4× Hifair III SuperMix Plus (Cat. #11142ES; Yeasen). Quantitative PCR was performed in a 20 µL reaction using Hieff UNICON Universal Blue qPCR SYBR Green Master Mix (Cat. #11184ES; Yeasen) on an ABI Q7 Fast Real‐Time PCR System (Applied Biosystems). Cycle threshold values were determined automatically by QuantStudio software under default parameters. β‐Actin mRNA (*ACTB*) served as the endogenous normalization control. Primer sequences were as follows:

*SLC2A5* Forward: 5′‐GAGCTGGCCCCTAAAAACCT‐3′
*SLC2A5* Reverse: 5′‐GAAGAAGGGCAGCAGAAGGA‐3′
*ACTB* forward: 5′‐CATGTACGTTGCTATCCAGGC‐3′
*ACTB* Reverse: 5′‐CTCCTTAATGTCACGCACGAT‐3′

### 4.18 Immunoblotting

Total cellular proteins were extracted from cells cultured in 10 cm dishes using high‐efficiency RIPA lysis buffer (Solarbio; Cat. #R0010, Beijing, China) supplemented with protease inhibitor cocktail (APExBIO; Cat. #K1007, Houston, USA) and 1 mm PMSF (Solarbio; Cat. #P0100). Cell lysates were incubated on ice for 30 min with intermittent vortexing, and then centrifuged at 13000 × *g* for 20 min at 4°C to remove cellular debris. Protein concentrations were measured by bicinchoninic acid protein assay kit and adjusted to 2 mg mL^−1^ with lysis buffer. 20 µL aliquots of each protein sample were combined with 5× SDS loading buffer (ABclonal; Cat. #RM00001, Wuhan, China) and denatured at 95°C for 10 min. The denatured proteins were then resolved by SDS‐PAGE on 10% polyacrylamide gels at 100 V for 90 min, then transferred to 0.45 µm PVDF membranes (Millipore; Cat. #IPVH00010, Billerica, USA) using a wet transfer system at 100 V for 60 min at 4°C. Membranes were blocked in 5% non‐fat dry milk (Bio‐Rad; Cat. #170‐6404, USA) in tris‐buffered saline with tween 20 (TBST) for 1 h at room temperature with gentle agitation. The blocked membranes were incubated with primary antibodies overnight at 4°C, followed by horseradish peroxidase‐conjugated secondary antibodies (1:5000) for 1 h at room temperature, with three 10 min washes in TBST after each incubation. Immunoreactive bands were visualized using ECL Chemiluminescent Substrate Detection Kit (Cat# K1200, APExBIO) and imaged on a ChemDoc MP Imaging system (Bio‐Rad).

### 4.19 Cell Viability Assay

U‐2932 WT or *SLC2A5*‐knockout cells were seeded at 2 × 10^4^ cells per well in 96‐well flat‐bottom microplates (Corning, USA) and allowed to recover in complete RPMI‐1640 medium at 37°C, 5% CO_2_ for 24 h. Subsequently, cells were maintained in glucose‐depleted RPMI‐1640 medium and treated with different concentrations of glucose, fructose, or 2,5‐AM for five consecutive days under standard culture conditions. Cell viability was assessed daily using the Cell Counting Kit‐8 (CCK‐8; APExBIO, Cat. #K1018, USA) according to the manufacturer's instructions. At each time point, 10 µL of CCK‐8 reagent was added to each well containing 100 µL of cell culture medium, followed by incubation for 2 h at 37°C. The absorbance was measured at 450 nm on a Synergy HTX multimode microplate reader (BioTek Instruments, USA).

### 4.20 Proliferation and Colony Formation Assays

The colony formation assay was performed using MethoCult H4230 methylcellulose‐based medium (Catalog #04430, StemCell Technologies Inc., Vancouver, Canada), according to the manufacturer's instructions. Briefly, single‐cell suspensions were mixed with the H4230 medium at a concentration of 1 × 10^4^ cells mL^−1^, and 500 µL aliquots were plated in triplicate into 24‐well ultra‐low‐attachment plates (Corning Costar, Cat. #3473). Cells were cultured at 37°C, 5% CO_2_, and 95% humidity for 14 d. Colonies—defined as aggregates of ≥50 cells—were counted and imaged using Operetta CLS high‐content system (PerkinElmer, UK). Colony formation efficiency (CFE) was calculated as (number of colonies formed / number of cells seeded) × 100%.

### 4.21 Fructose Uptake Measurements

WT and *SLC2A5*‐knockout U‐2932 cells were recovered in complete medium for 24 h and then seeded into 6‐well plates at a density of 1 × 10^6^ cells per well in 3 mL glucose‐free medium. After 18 h of incubation, fructose was added to the culture medium at final concentrations of 0 or 10 mm, and cells were cultured for an additional 18 h. Cells were then counted, and the conditioned media were collected to assess fructose consumption. Fructose concentrations were measured using a fructose assay kit (G0530F, Grace Biotechnology, China) according to the manufacturer's instructions. Absorbance was measured at 340 nm using a microplate reader. Fructose consumption was normalized to cell number.

### 4.22 Orthotopic Mouse Model of PCNSL

Luciferase‐expressing U‐2932 cells (4×10^5^ cells in 5 µL PBS) were stereotactically injected into the prefrontal lobe of 5‐week‐old male NSIG mice (*n* = 5 per group; HFK Bioscience, Beijing, China) under isoflurane anesthesia using a Hamilton syringe with a 26‐gauge needle. The needle was positioned in the prefrontal cortex at coordinates +1.0 mm anterior and +2.5 mm lateral to bregma, lowered to a depth of 4.0 mm from the skull surface, then partially withdrawn to 3.0 mm before cell injection. To assess the therapeutic potential of 2,5‐anhydro‐D‐mannitol (2,5‐AM; Cayman Chemical), treatment was initiated 7 d post‐implantation to allow for tumor establishment. Mice received daily intraperitoneal injections of 2,5‐AM at either 75 mg kg^−1^ or 200 mg kg^−1^ (in 200 µL sterile PBS) until experimental endpoints. Tumor burden was serially monitored every 7 d via bioluminescent imaging (IVIS Spectrum, PerkinElmer) following intraperitoneal administration of 150 mg kg^−1^ VivoGlo Luciferin (Promega, USA) 10 min prior to imaging.

To assess the impact of fructose‐uptake blockade via *SLC2A5* knockout on PCNSL progression, luciferase‐expressing U‐2932 WT and *SLC2A5*‐knockout cells (4 × 10^5^ cells in 5 µL) were stereotactically implanted into the prefrontal cortex of NSIG mice as described above. Tumor burden was evaluated by bioluminescent imaging on days 3, 14, and 21 post‐implantations without therapeutic intervention. Parallel cohorts (*n* = 5 per group) were monitored for survival, with endpoints defined by onset of neurological deficits (lethargy, circling behavior), >20% body weight loss, or other signs of morbidity.

Mice were maintained in specific pathogen‐free conditions with environmental parameters maintained as follows: relative humidity 50–65%, temperature 23 ± 2°C, and a controlled 12‐hour light/dark cycle (lights on at 7:00 AM). Animals were group‐housed (3‐5 mice per cage) in individually ventilated cages containing standard corn cob bedding, with ad libitum access to autoclaved rodent chow (HFK Bioscience, Beijing) and sterile drinking water. The animal use in this study was approved by Animal Ethics and Welfare Committee of Tianjin Huanhu Hospital (Approval No. HHLL–2025‐065). Tumor burden was monitored twice weekly and never exceeded 10% of body weight or 1.5 cm in any dimension, in accordance with Institutional Animal Care and Use Committee‐specified humane endpoints.

### 4.23 IHC Staining

The IHC staining was carried out following the manufacturer's protocol using the standard staining kit provided with the Leica automated system. Briefly, paraffin‐embedded tumor tissue sections were first heated at 68°C for 15 min and subsequently processed on the BOND‐III automated stainer (Leica, Germany). Antigen retrieval was conducted using EDTA buffer (pH 6.0) for 5 min. Endogenous peroxidase activity was blocked by incubating with 3% hydrogen peroxide for 5 min at room temperature. The sections were then incubated with either primary antibody against ubiquitinated histone H2B (uH2B; Cell Signaling Technology) or IgG (as negative control) for 15 min at room temperature. Following incubation with the secondary antibody for 8 min at room temperature, the reaction was visualized using 3,3'‐diaminobenzidine chromogen, and cell nuclei were counterstained with hematoxylin. Finally, stained sections were examined under an Olympus BX63 upright microscope (Japan).

### 4.24 Quantification and Statistical Analysis

Statistical details, including sample size (*N*), mean values, and statistical significance, are provided in the text, figure legends, or methods section. Error bars in the figures represent either the standard error of the mean (SEM) or standard deviation (SD), derived from independent experiments, independent biological replicates, or independent samples. All statistical analyses were conducted using GraphPad Prism software unless otherwise specified, with detailed descriptions of the statistical methods provided in the figure legends or methods section. *P* values (*p < 0.05, **p < 0.01, ***p < 0.001, ****p < 0.0001, n.s. not significant) are indicated in the respective figures. A *p* value < 0.05 was considered statistically significant.

## Author Contributions

Conceptualization: Q.L., G.A., and Y.L. Methodology: Q.W., Q.Z., W.Y., L.S., Y.C., B.L., G.H., F.Z., K.P., M.Z., X.Z., Q.L., G.A., and Y.L. Investigation: Q.W., Q.Z., W.Y., L.S., Y.C., B.L., G.H., F.Z., K.P., and M.Z. writing – original draft: Q.W., Q.Z., W.Y., L.S., Q.L., G.A., and Y.L. Writing – review & editing: Q.W., Q.Z., W.Y., L.S., Q.L., G.A., and Y.L. Funding acquisition, Q.L., G.A., and Y.L. Supervision: Q.W., X.Z., Q.L., G.A., and Y.L.

## Conflicts of Interest

The authors declare no conflicts of interest.

## Supporting information




**Supporting File 1**: advs76316‐sup‐0001‐SuppMat.docx.

**Supporting File 2**: advs76316‐sup‐0002‐TableS1.xlsx.

**Supporting File 3**: advs76316‐sup‐0003‐TableS2.xls.

**Supporting File 4**: advs76316‐sup‐0004‐TableS3.xlsx.

## Data Availability

The raw scRNA‐seq and scBCR‐seq data are available Genome Sequence Archive (GSA) for Human in the BIG Data Center (https://ngdc.cncb.ac.cn/gsa‐human/) under accession number HRA011982. Processed data and code for generating the processed data are available upon request.

## References

[1] M. Roschewski and D. J. Hodson , “Diffuse Large B‐Cell Lymphoma Involving the central Nervous System: Biologic Rationale for Targeted Therapy,” Haematologica 109, no. 2 (2024): 388–400, 10.3324/haematol.2021.278613. PMC10828633 37706315

[2] A. J. M. Ferreri , T. Calimeri , and K. Cwynarski , “Primary central Nervous System Lymphoma,” Nature Reviews Disease Primers 9, no. 1 (2023): 29, 10.1038/s41572-023-00439-0. PMC10637780 37322012

[3] I. Hernández‐Verdin , E. Kirasic , and K. Wienand , “Molecular and Clinical Diversity in Primary central Nervous System Lymphoma,” Annals of Oncology 34, no. 2 (2023): 186–199, 10.1016/j.annonc.2022.11.002. 36402300

[4] J. Radke , N. Ishaque , R. Koll , et al., “The Genomic and Transcriptional Landscape of Primary central Nervous System Lymphoma,” Nature Communications 13, no. 1 (2022): 2558, 10.1038/s41467-022-30050-y. PMC9091224 35538064

[5] H. Shi , X. Sun , and Y. Wu , “Targeting the Tumor Microenvironment in Primary central Nervous System Lymphoma: Implications for Prognosis,” Journal of Clinical Neuroscience 124 (2024): 36–46, 10.1016/j.jocn.2024.04.009. 38642434

[6] C. Chang , C. H. Lin , A. L. Cheng , L. J. Medeiros , and K. C. Chang , “Primary central Nervous System Diffuse Large B‐Cell Lymphoma Has Poorer Immune Cell Infiltration and Prognosis than Its Peripheral Counterpart,” Histopathology 67, no. 5 (2015): 625–635, 10.1111/his.12706. 25829022

[7] N. Liu , C. Jiang , and X. Yao , “Single‐Cell Landscape of Primary central Nervous System Diffuse Large B‐Cell Lymphoma,” Cell Discovery 9, no. 1 (2023): 55, 10.1038/s41421-023-00559-7. PMC10261103 37308475

[8] M. Heming , S. Haessner , and J. Wolbert , “Intratumor Heterogeneity and T Cell Exhaustion in Primary CNS Lymphoma,” Genome Medicine 14, no. 1 (2022): 109, 10.1186/s13073-022-01110-1. PMC9509601 36153593

[9] S. Yoshida , K. Morii , M. Watanabe , and T. Saito , “Characteristic Features of Malignant Lymphoma with central Nervous System Involvement,” Surgical Neurology 53, no. 2 (2000): 163–167, 10.1016/S0090-3019(99)00186-X. 10713195

[10] J. W. Taylor , E. P. Flanagan , and B. P. O'Neill , “Primary Leptomeningeal Lymphoma,” Neurology 81, no. 19 (2013): 1690–1696, 10.1212/01.wnl.0000435302.02895.f3. PMC3812109 24107866

[11] J. A. Kim , S. J. kim , and I.‐G. Do , “Hypoxia‐Associated Protein Expression in Primary Central Nervous System Diffuse Large B‐Cell Lymphoma: Does It Predict Prognosis?,” Leukemia & Lymphoma 52, no. 2 (2011): 205–213, 10.3109/10428194.2010.542261. 21281236

[12] H. W. King , K. L. Wells , and Z. Shipony , “Integrated Single‐Cell Transcriptomics and Epigenomics Reveals Strong Germinal Center–associated Etiology of Autoimmune Risk Loci,” Science Immunology 6, no. 64 (2021): abh3768, 10.1126/sciimmunol.abh3768. PMC8859880 34623901

[13] A. R. Thompsett , D. W. Ellison , F. K. Stevenson , and D. Zhu , “VH Gene Sequences from Primary Central Nervous System Lymphomas Indicate Derivation from Highly Mutated Germinal Center B Cells with Ongoing Mutational Activity,” Blood 94, no. 5 (1999): 1738–1746, 10.1182/blood.V94.5.1738. 10477699

[14] J. Liu , Y. Wang , Y. Liu , et al., “Immunohistochemical Profile and Prognostic Significance in Primary central Nervous System Lymphoma: Analysis of 89 Cases,” Oncology letters 14, no. 5 (2017): 5505–5512. 10.3892/ol.2017.6893 PMC5656017 29113178

[15] K. Kluckova , A. D'Avola , and J. C. Riches , “Advances in Understanding of Metabolism of B‐Cell Lymphoma: Implications for Therapy,” Cancers 14, no. 22 (2022): 5552, 10.3390/cancers14225552. PMC9688663 36428647

[16] W. Zhou , J. Wen , and F. Hua , “18 F‐FDG PET/CT in Immunocompetent Patients with Primary central Nervous System Lymphoma: Differentiation from Glioblastoma and Correlation with DWI,” European Journal of Radiology 104 (2018): 26–32, 10.1016/j.ejrad.2018.04.020. 29857862

[17] K. Tateishi , Y. Miyake , and M. Kawazu , “A Hyperactive RelA/p65‐Hexokinase 2 Signaling Axis Drives Primary Central Nervous System Lymphoma,” Cancer Research 80, no. 23 (2020): 5330–5343, 10.1158/0008-5472.CAN-20-2425. 33067267

[18] L. E. Nigrovic , A. A. Kimia , S. S. Shah , and M. I. Neuman , “Relationship between Cerebrospinal Fluid Glucose and Serum Glucose,” New England Journal of Medicine 366, no. 6 (2012): 576–578, 10.1056/NEJMc1111080. 22316468

[19] M. A. Bentsen , Z. Mirzadeh , and M. W. Schwartz , “Revisiting How the Brain Senses Glucose—And Why,” Cell Metabolism 29, no. 1 (2019): 11–17, 10.1016/j.cmet.2018.11.001. PMC6326855 30527741

[20] B. J. Scott , V. C. Douglas , T. Tihan , J. L. Rubenstein , and S. A. Josephson , “A Systematic Approach to the Diagnosis of Suspected central Nervous System Lymphoma,” JAMA Neurology 70, no. 3 (2013): 311–319, 10.1001/jamaneurol.2013.606. PMC4135394 23319132

[21] C. B. Steen , B. A. Luca , and M. S. Esfahani , “The Landscape of Tumor Cell States and Ecosystems in Diffuse Large B Cell Lymphoma,” Cancer Cell 39, no. 10 (2021): 1422–1437.e10, 10.1016/j.ccell.2021.08.011. PMC9205168 34597589

[22] T. Roider , J. Seufert , and A. Uvarovskii , “Dissecting Intratumour Heterogeneity of Nodal B‐Cell Lymphomas at the Transcriptional, Genetic and Drug‐Response Levels,” Nature Cell Biology 22, no. 7 (2020): 896–906, 10.1038/s41556-020-0532-x. 32541878

[23] K. Tao , X. Wang , and X. Tian , “Relapsed Primary Central Nervous System Lymphoma: Current Advances,” Frontiers in Oncology 11 (2021): 649789, 10.3389/fonc.2021.649789. PMC8118624 33996566

[24] N. Babst , L. K. Isbell , and F. Rommel , “CXCR4, CXCR5 and CD44 May Be Involved in Homing of Lymphoma Cells into the Eye in a Patient Derived Xenograft Homing Mouse Model for Primary Vitreoretinal Lymphoma,” International Journal of Molecular Sciences 23, no. 19 (2022): 11757, 10.3390/ijms231911757. PMC9569795 36233057

[25] Y. Song , W. Zhang , and L. Zhang , “Cerebrospinal Fluid IL‐10 and IL‐10/IL‐6 as Accurate Diagnostic Biomarkers for Primary Central Nervous System Large B‐Cell Lymphoma,” Scientific Reports 6 (2016): 38671, 10.1038/srep38671. PMC5141427 27924864

[26] Y. Huang , V. Mohanty , and M. Dede , “Characterizing Cancer Metabolism from Bulk and Single‐Cell RNA‐seq Data Using METAFlux,” Nature Communications 14, no. 1 (2023): 4883, 10.1038/s41467-023-40457-w. PMC10423258 37573313

[27] A. Reddy , J. Zhang , and N. S. Davis , “Genetic and Functional Drivers of Diffuse Large B Cell Lymphoma,” Cell 171, no. 2 (2017): 481–494.e15, 10.1016/j.cell.2017.09.027. PMC5659841 28985567

[28] G. Pizzolato , H. Kaminski , and M. Tosolini , “Single‐Cell RNA Sequencing Unveils the Shared and the Distinct Cytotoxic Hallmarks of human TCRVδ1 and TCRVδ2 Γδ T Lymphocytes,” Proceedings of the National Academy of Sciences 116, no. 24 (2019): 11906–11915, 10.1073/pnas.1818488116. PMC6576116 31118283

[29] N. L. de Vries , J. van de Haar , V. Veninga , et al., “Gammadelta T Cells Are Effectors of Immunotherapy in Cancers with HLA Class I Defects,” Nature 613, no. 7945 (2023): 743–750. 10.1038/s41586-022-05593-1 PMC9876799 36631610

[30] C. Rancan , M. Arias‐Badia , and P. Dogra , “Exhausted Intratumoral Vδ2− Γδ T Cells in human Kidney Cancer Retain Effector Function,” Nature Immunology 24, no. 4 (2023): 612–624, 10.1038/s41590-023-01448-7. PMC10063448 36928415

[31] H. Li , A. M. van der Leun , and I. Yofe , “Dysfunctional CD8 T Cells Form a Proliferative, Dynamically Regulated Compartment within Human Melanoma,” Cell 176, no. 4 (2019): 775–789.e18, 10.1016/j.cell.2018.11.043. PMC7253294 30595452

[32] L. Zheng , S. Qin , and W. Si , “Pan‐Cancer Single‐Cell Landscape of Tumor‐Infiltrating T Cells,” Science 374, no. 6574 (2021): abe6474, 10.1126/science.abe6474. 34914499

[33] E. Zhao , T. Maj , and I. Kryczek , “Cancer Mediates Effector T Cell Dysfunction by Targeting microRNAs and EZH2 via Glycolysis Restriction,” Nature Immunology 17, no. 1 (2016): 95–103, 10.1038/ni.3313. PMC4684796 26523864

[34] A. R. Pombo Antunes , I. Scheyltjens , F. Lodi , et al., “Single‐Cell Profiling of Myeloid Cells in Glioblastoma across Species and Disease Stage Reveals Macrophage Competition and Specialization,” Nature Neuroscience 24, no. 4 (2021): 595–610. 10.1038/s41593-020-00789-y 33782623

[35] M. Olah , V. Menon , and N. Habib , “Single Cell RNA Sequencing of human Microglia Uncovers a Subset Associated with Alzheimer's Disease,” Nature Communications 11, no. 1 (2020): 6129, 10.1038/s41467-020-19737-2. PMC7704703 33257666

[36] W. Wang , T. Li , and Y. Cheng , “Identification of Hypoxic Macrophages in Glioblastoma with Therapeutic Potential for Vasculature Normalization,” Cancer Cell 42, no. 5 (2024): 815–832.e12, 10.1016/j.ccell.2024.03.013. 38640932

[37] R. Sankowski , C. Böttcher , and T. Masuda , “Mapping Microglia States in the Human Brain through the Integration of High‐Dimensional Techniques,” Nature Neuroscience 22, no. 12 (2019): 2098–2110, 10.1038/s41593-019-0532-y. 31740814

[38] S. Müller , G. Kohanbash , and S. J. Liu , “Single‐Cell Profiling of human Gliomas Reveals Macrophage Ontogeny as a Basis for Regional Differences in Macrophage Activation in the Tumor Microenvironment,” Genome Biology 18, no. 1 (2017): 234, 10.1186/s13059-017-1362-4. PMC5738907 29262845

[39] N. Abdelfattah , P. Kumar , and C. Wang , “Single‐Cell Analysis of human Glioma and Immune Cells Identifies S100A4 as an Immunotherapy Target,” Nature Communications 13, no. 1 (2022): 767, 10.1038/s41467-022-28372-y. PMC8828877 35140215

[40] Y. Otani , Y. Yamaguchi , and Y. Sato , “PLD4 Is Involved in Phagocytosis of Microglia: Expression and Localization Changes of PLD4 Are Correlated with Activation State of Microglia,” PLoS One 6, no. 11 (2011): 27544, 10.1371/journal.pone.0027544. PMC3216956 22102906

[41] M. J. Grimshaw , T. Hagemann , A. Ayhan , C. E. Gillett , C. Binder , and F. R. Balkwill , “A Role for Endothelin‐2 and Its Receptors in Breast Tumor Cell Invasion,” Cancer Research 64, no. 7 (2004): 2461–2468, 10.1158/0008-5472.CAN-03-1069. 15059899

[42] A. Mantovani , A. Sica , S. Sozzani , P. Allavena , A. Vecchi , and M. Locati , “The Chemokine System in Diverse Forms of Macrophage Activation and Polarization,” Trends in Immunology 25, no. 12 (2004): 677–686, 10.1016/j.it.2004.09.015. 15530839

[43] A. T. Henze and M. Mazzone , “The Impact of Hypoxia on Tumor‐Associated Macrophages,” Journal of Clinical Investigation 126, no. 10 (2016): 3672–3679, 10.1172/JCI84427. PMC5096805 27482883

[44] M. Tausendschön , M. Rehli , and N. Dehne , “Genome‐Wide Identification of Hypoxia‐Inducible Factor‐1 and ‐2 Binding Sites in Hypoxic human Macrophages Alternatively Activated by IL‐10,” Biochimica et Biophysica Acta (BBA)—Gene Regulatory Mechanisms 1849, no. 1 (2015): 10–22, 10.1016/j.bbagrm.2014.10.006. 25450522

[45] T. Liu , J. A. Ortiz , and L. Taing , “Cistrome: an Integrative Platform for Transcriptional Regulation Studies,” Genome Biology 12, no. 8 (2011): R83, 10.1186/gb-2011-12-8-r83. PMC3245621 21859476

[46] M. Mulazzani , S. P. Frassle , I. von Mucke‐Heim , et al., “Long‐Term in Vivo Microscopy of CAR T Cell Dynamics during Eradication of CNS Lymphoma in Mice,” Proceedings of the National Academy of Sciences 116, no. 48 (2019): 24275–24284, 10.1073/pnas.1903854116. PMC6883823 31712432

[47] X. Zhou , M. Mulazzani , I. A. von Mucke‐Heim , et al., “The Role of BAFF‐R Signaling in the Growth of Primary Central Nervous System Lymphoma,” Frontiers in Oncology 10 (2020): 682, 10.3389/fonc.2020.00682. PMC7266954 32528875

[48] C. Chen , Z. Zhang , and C. Liu , “ATF4‐Dependent Fructolysis Fuels Growth of Glioblastoma Multiforme,” Nature Communications 13, no. 1 (2022): 6108, 10.1038/s41467-022-33859-9. PMC9573865 36245009

[49] M. B. Vos , J. E. Kimmons , C. Gillespie , J. Welsh , and H. M. Blanck , “Dietary Fructose Consumption among US Children and Adults: the Third National Health and Nutrition Examination Survey,” Medscape Journal of Medicine 10, no. 7 (2008): 160. PMC2525476 18769702

[50] H. Hui , D. Huang , D. McArthur , N. Nissen , L. G. Boros , and A. P. Heaney , “Direct Spectrophotometric Determination of Serum Fructose in Pancreatic Cancer Patients,” Pancreas 38, no. 6 (2009): 706–712, 10.1097/MPA.0b013e3181a7c6e5. 19506535

[51] Y. Weng , J. Zhu , Z. Chen , J. Fu , and F. Zhang , “Fructose Fuels Lung Adenocarcinoma through GLUT5,” Cell Death & Disease 9, no. 5 (2018): 557, 10.1038/s41419-018-0630-x. PMC5945656 29748554

[52] W.‐L. Chen , Y.‐Y. Wang , and A. Zhao , “Enhanced Fructose Utilization Mediated by SLC2A5 Is a Unique Metabolic Feature of Acute Myeloid Leukemia with Therapeutic Potential,” Cancer Cell 30, no. 5 (2016): 779–791, 10.1016/j.ccell.2016.09.006. PMC5496656 27746145

[53] C. Su , H. Li , and W. Gao , “GLUT5 increases Fructose Utilization and Promotes Tumor Progression in Glioma,” Biochemical and Biophysical Research Communications 500, no. 2 (2018): 462–469, 10.1016/j.bbrc.2018.04.103. 29660339

[54] C. Wang , L. Wang , and Q. Zhao , “Exploring Fructose Metabolism as a Potential Therapeutic Approach for Pancreatic Cancer,” Cell Death & Differentiation 31, no. 12 (2024): 1625–1635, 10.1038/s41418-024-01394-3. PMC11618635 39406919

[55] Q. Zhao , B. Han , and L. Wang , “AKR1B1‐Dependent Fructose Metabolism Enhances Malignancy of Cancer Cells,” Cell Death & Differentiation 31, no. 12 (2024): 1611–1624, 10.1038/s41418-024-01393-4. PMC11618507 39406918

[56] H. Yan , Z. Wang , and D. Teng , “Hexokinase 2 Senses Fructose in Tumor‐Associated Macrophages to Promote Colorectal Cancer Growth,” Cell Metabolism 36, no. 11 (2024): 2449–2467.e6, 10.1016/j.cmet.2024.10.002. 39471815

[57] B. Dura , J.‐Y. Choi , and K. Zhang , “scFTD‐seq: Freeze‐Thaw Lysis Based, Portable Approach toward Highly Distributed Single‐Cell 3′ mRNA Profiling,” Nucleic Acids Research 47, no. 3 (2019): 16, 10.1093/nar/gky1173. PMC6379653 30462277

[58] A. T. L. Lun , S. Riesenfeld , T. Andrews , and T. P. Dao , “EmptyDrops: Distinguishing Cells from Empty Droplets in Droplet‐Based Single‐Cell RNA Sequencing Data,” Genome Biology 20, no. 1 (2019): 63, 10.1186/s13059-019-1662-y. PMC6431044 30902100

[59] Y. Hao , S. Hao , and E. Andersen‐Nissen , “Integrated Analysis of Multimodal Single‐Cell Data,” Cell 184, no. 13 (2021): 3573–3587.e29, 10.1016/j.cell.2021.04.048. PMC8238499 34062119

[60] S. Yang , S. E. Corbett , and Y. Koga , “Decontamination of Ambient RNA in Single‐Cell RNA‐Seq with DecontX,” Genome Biology 21, no. 1 (2020): 57, 10.1186/s13059-020-1950-6. PMC7059395 32138770

[61] T. Tickle , I. Tirosh , C. Georgescu , M. Brown , and B. Haas . inferCNV of the Trinity CTAT Project (Broad Institute of MIT and Harvard, 2019).

[62] S. Kurtenbach , A. M. Cruz , D. A. Rodriguez , M. A. Durante , and J. W. Harbour , “Uphyloplot2: Visualizing Phylogenetic Trees from Single‐Cell RNA‐seq Data,” BMC Genomics 22, no. 1 (2021): 419, 10.1186/s12864-021-07739-3. PMC8180062 34090344

[63] L. Yerly , M. Andreatta , J. Garnica , et al., “Wounding Triggers Invasive Progression in Human Basal Cell Carcinoma,” *bioRxiv* (2025): 2024.05.31.596823, 10.1101/2024.05.31.596823.

[64] N. Borcherding , N. L. Bormann , and G. Kraus , “scRepertoire: An R‐Based Toolkit for Single‐Cell Immune Receptor Analysis,” F1000Research 9 (2020): 47, 10.12688/f1000research.22139.1. PMC7400693 32789006

[65] G. La Manno , R. Soldatov , A. Zeisel , et al., “RNA Velocity of Single Cells,” Nature 560, no. 7719 (2018): 494–498. 10.1038/s41586-018-0414-6 PMC6130801 30089906

[66] V. Bergen , M. Lange , S. Peidli , F. A. Wolf , and F. J. Theis , “Generalizing RNA Velocity to Transient Cell States through Dynamical Modeling,” Nature Biotechnology 38, no. 12 (2020): 1408–1414, 10.1038/s41587-020-0591-3. 32747759

[67] X. Qiu , A. Hill , J. Packer , D. Lin , Y. A. Ma , and C. Trapnell , “Single‐Cell mRNA Quantification and Differential Analysis with Census,” Nature Methods 14, no. 3 (2017): 309–315, 10.1038/nmeth.4150. PMC5330805 28114287

