# Supplementary material for: Single‐Cell Profiling Identifies SLC2A5‐Mediated Fructose Metabolism as a Vulnerability in Primary CNS Lymphoma
Source: Adv Sci (Weinh). 2026 Jun 26:e76316. Online ahead of print. doi: 10.1002/advs.76316 (PMC13336764; doi:10.1002/advs.76316)
Supplement: Supplementary file 1 — Supporting File 1: advs76316‐sup‐0001‐SuppMat.docx. [file ADVS-9999-e76316-s001.docx]

**
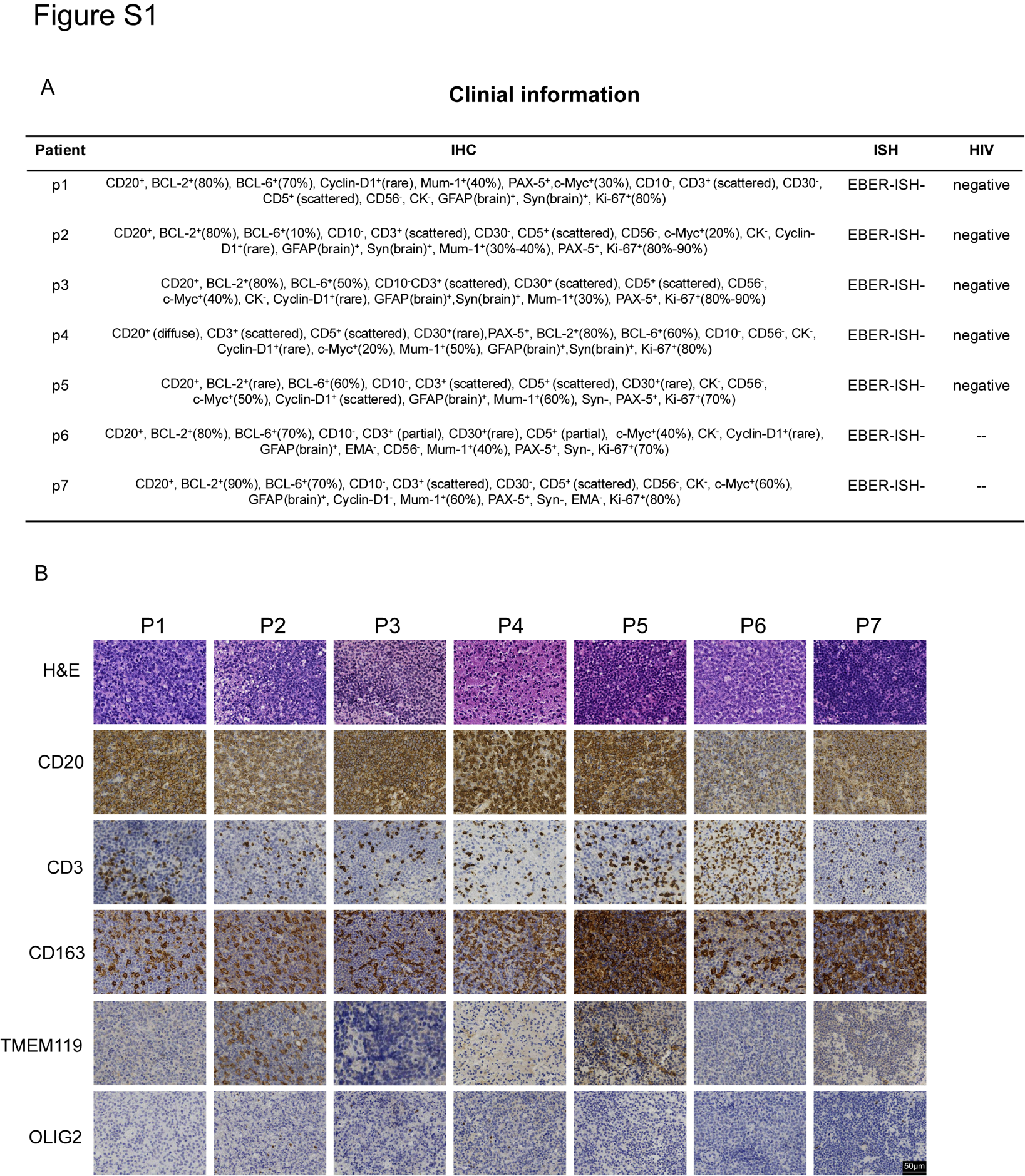
Supplemental Figures and Legends**

**Supplementary Figure 1. Clinical information and representative H&E and IHC staining**

(A) Table summarizing the clinical information of patients, including the reports of immunohistochemistry (IHC), in-situ hybridization (ISH) of Epstein-Barr virus-encoded small RNA (EBER), HIV infection status.

(B) Representative H&E staining and IHC staining images of CD20, CD3, CD163, TMEM119 and OLIG2 across all seven patients.

**
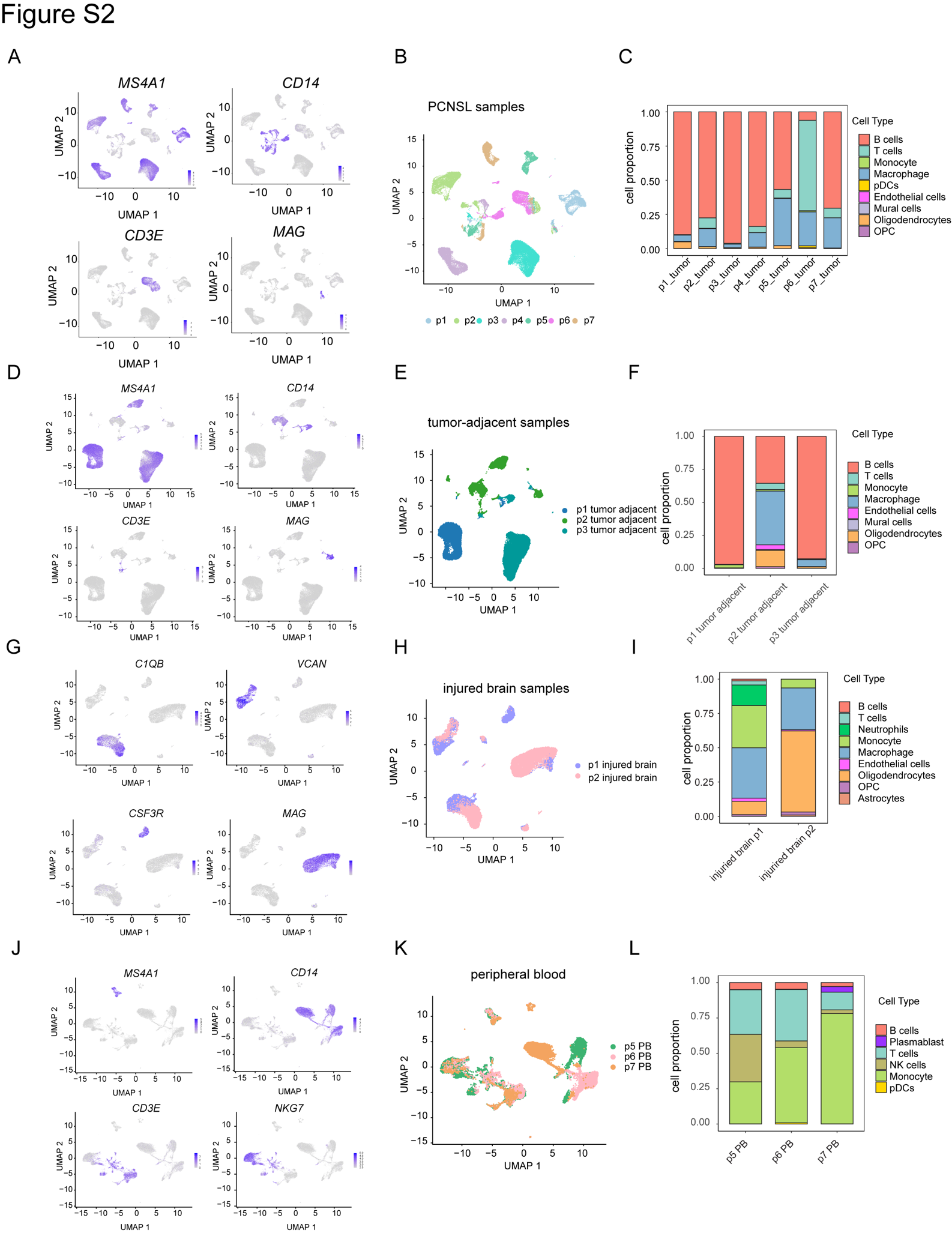
**

**Supplementary Figure 2. Single-cell landscape of PCNSL and non-tumor controls**

(A) Uniform manifold approximation and projection (UMAP) plots showing expression levels of selected marker genes for each major cell type in primary central nervous system lymphoma (PCNSL) samples.

(B) UMAP visualization of single-cell transcriptomes from PCNSL smaples. Each dot represents a single cell, colored by patient identities.

(C) The frequencies of annotated cell populations in tumor samples from each patient.

(D) UMAP plots showing expression levels of selected marker genes for each major cell type in tumor-adjacent samples.

(E) UMAP visualization of single-cell transcriptomes from tumor-adjacent tissues. Each dot represents a single cell, colored patient identities.

(F) The frequencies of annotated cell populations in tumor-adjacent samples from each patient.

(G) UMAP plots showing expression levels of selected marker genes for each major cell type in injuried brain tissues.

(H) UMAP visualization of single-cell transcriptomes from injured brain tissues. Each dot represents a single cell, colored by patient identities.

(I) The frequencies of annotated cell populations in injured brain samples from each patient.

(J) UMAP plots showing expression levels of selected marker genes for each major cell type in pereheral blood (PB).

(K) UMAP visualization of single-cell transcriptomes from PB. Each dot represents a single cell, colored by patient identities.

(L) The frequencies of annotated cell populations in PB samples from each patient.

**
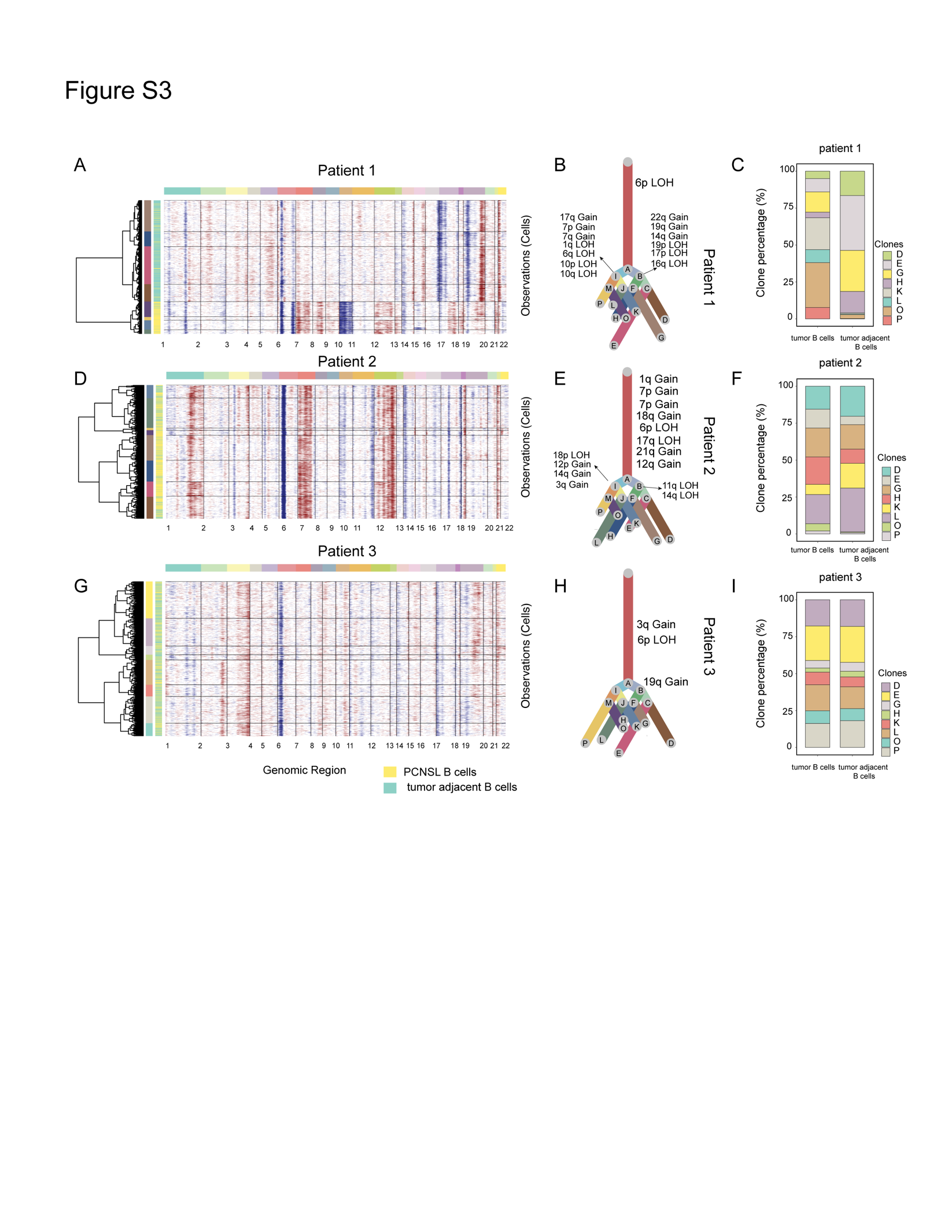
**

**Supplementary Figure 3. Subclonal architecture of PCNSL inferred from CNV analysis**

(A) Heatmap showing copy number variations（CNVs）inferred by inferCNV in B cells from PCNSL tumor samples (yellow) and tumor-adjacent tissues (green) of Patient1. Red indicates chromosomal amplifications; blue indicates deletions.

(B) Phylogenetic tree of patient1 constructed from inferCNV results using UPhyloplot2. The red branch represents the evolutionary transition from normal to malignant cells. Each node labeled with a different letter indicates a distinct tumor subclone. Branch length reflects the number of cells within the corresponding subclone.

(C) The percentages of distinct B cell subclones, identified based on CNV profiles, in PCNSL tumor and tumor-adjacent samples from patient1.

(D) Heatmap showing CNVs inferred by inferCNV in B cells from PCNSL tumor samples (yellow) and tumor-adjacent tissues (green) of patient2.

(E) Phylogenetic tree of patient2 constructed from inferCNV results using UPhyloplot2.

(F) The percentages of distinct B cell subclones, identified based on CNV profiles, in PCNSL tumor and tumor-adjacent samples from patient2.

(G) Heatmap showing CNVs inferred by inferCNV in B cells from PCNSL tumor samples (yellow) and tumor-adjacent tissues (green) of patient3.

(H) Phylogenetic tree of patient3 constructed from inferCNV results using UPhyloplot2.

(I) The percentages of distinct B cell subclones, identified based on CNV profiles, in PCNSL tumor and tumor-adjacent samples from patient3.

**
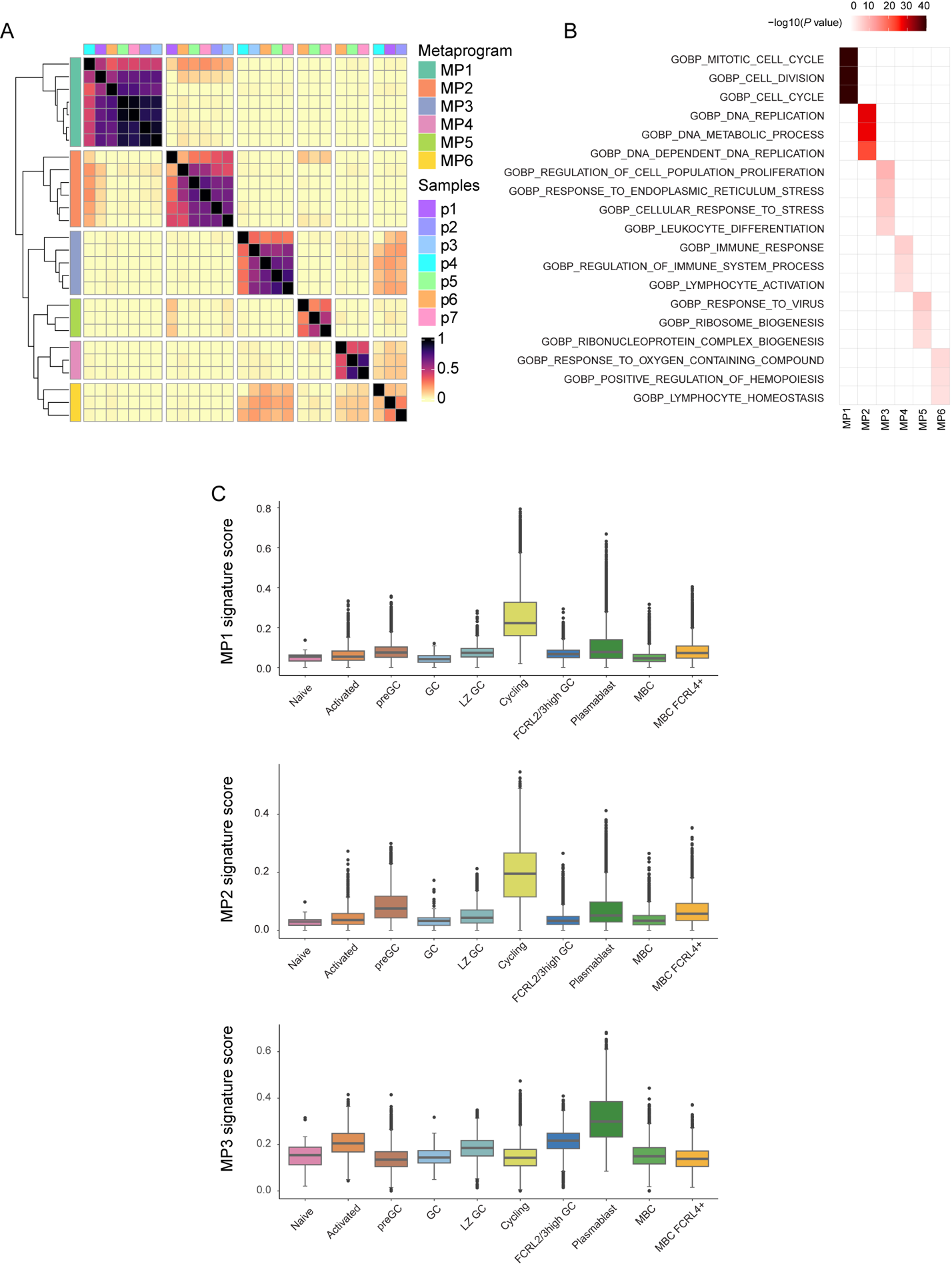
**

**Supplementary Figure 4. Meta-programs expressed in PCNSL B cells**

(A) Heatmap showing meta-programs identified in PCNSL B cells. Colors represent pairwise similarity (Jaccard index) between individual gene programs across different patients. Gene programs are hierarchically clustered and grouped into distinct meta-programs (MPs).

(B) Biological pathways significantly enriched in each identified MP.

(C) Scores of MP1 (top), MP2 (middle), and MP3 (bottom) across different B cell subtypes in PCNSL.

**
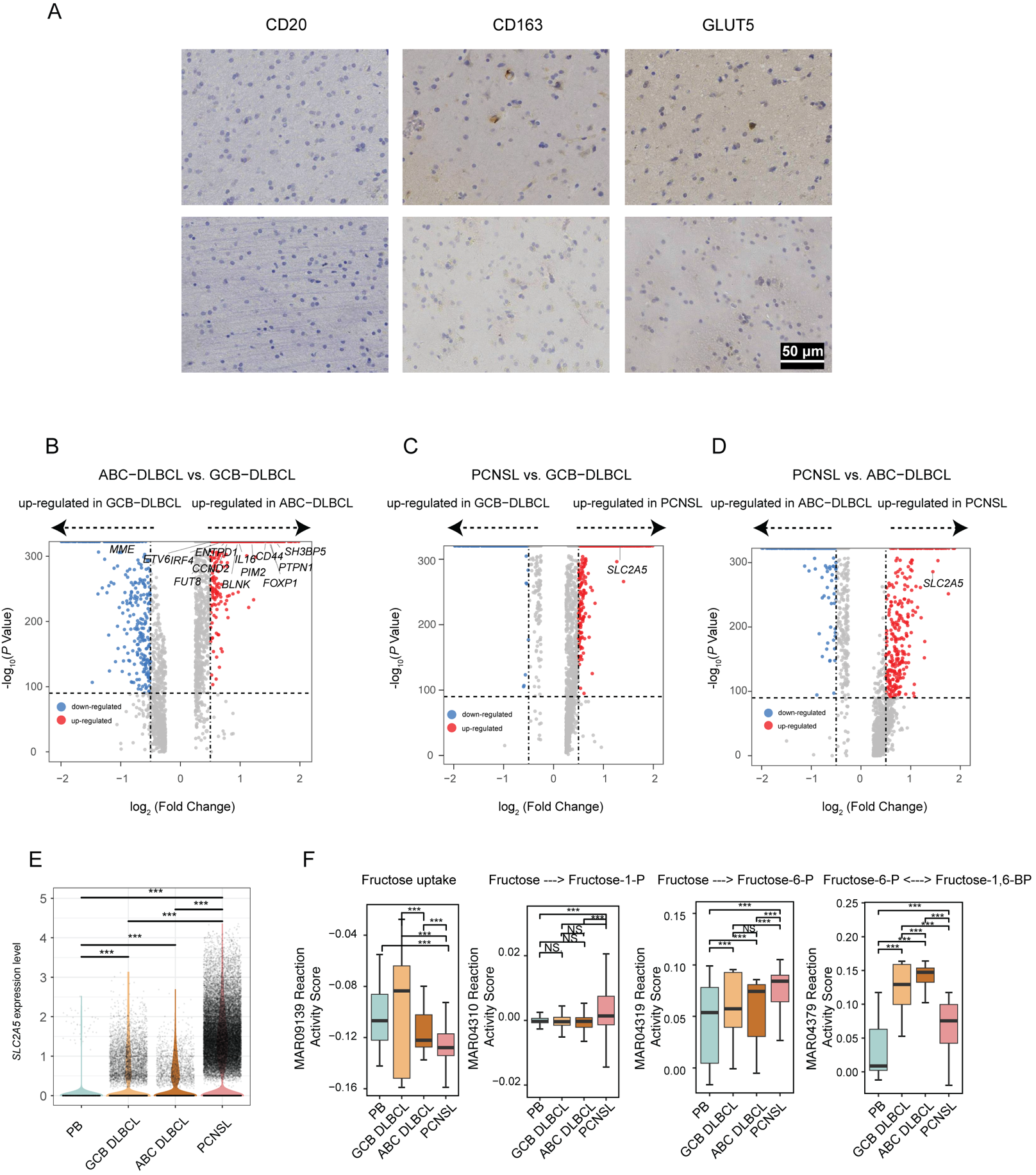
**

**Supplementary Figure 5. Fructose metabolism is upregulated in PCNSL compared to GCB-DLBCL and ABC-DLBCL**

(A) IHC staining of CD20, CD163 and GLUT5 in injured brain tissues. Injured brain tissues with minimal B-cell infiltrating were used as negative controls. Two representive views show that regions lacking CD20 and CD163 expression also exhibit negative GLUT5 expression.

(B) Volcano plot showing differentially expressed genes in B cells between ABC-DLBCL and GCB-DLBCL samples. Single-cell transcriptomic data of DLBCL were obtained from Steen et al. and Roider et al. Three patients with patient IDs DLBCL002, DLBCL008, and DLBCL111 from the original studies were grouped as ABC-DLBCL, and three patients with patient IDs DLBCL007, DLBCL1, and DLBCL2 were grouped as GCB-DLBCL.Genes with –log_10_(*p* value) > 90 and log_2_(fold change) > 0.5 are highlighted in red, and genes with –log_10_(*p* value) > 90 and log_2_(fold change) < –0.5 are highlighted in blue.

(C) Volcano plot showing differentially expressed genes in B cells between PCNSL and GCB-DLBCL samples. Genes with –log_10_(*p* value) > 90 and log_2_(fold change) > 0.5 are highlighted in red, and genes with –log_10_(*p* value) > 90 and log_2_(fold change) < –0.5 are highlighted in blue.

(D) Volcano plot showing differentially expressed genes in B cells between PCNSL and ABC-DLBCL samples. Genes with –log_10_(*p* value) > 90 and log_2_(fold change) > 0.5 are highlighted in red, and genes with –log_10_(*p* value) > 90 and log_2_(fold change) < –0.5 are highlighted in blue.

(E) *SLC2A5* expression levels across PB, DLBCL and PCNSL samples, with *** indicating *p* < 0.001.

(F) Inferred metabolic fluxes of fructose metabolism in B cells from PB, GCB-DLCBL, ABC-DLBCL and PCNSL, estimated using METAFlux. The y-axis represents flux scores calculated from METAFlux for each reaction. Differences in flux scores among groups were assessed using the Wilcoxon test, with *** indicating *p* < 0.001 and NS indicating non-significant differences.

**
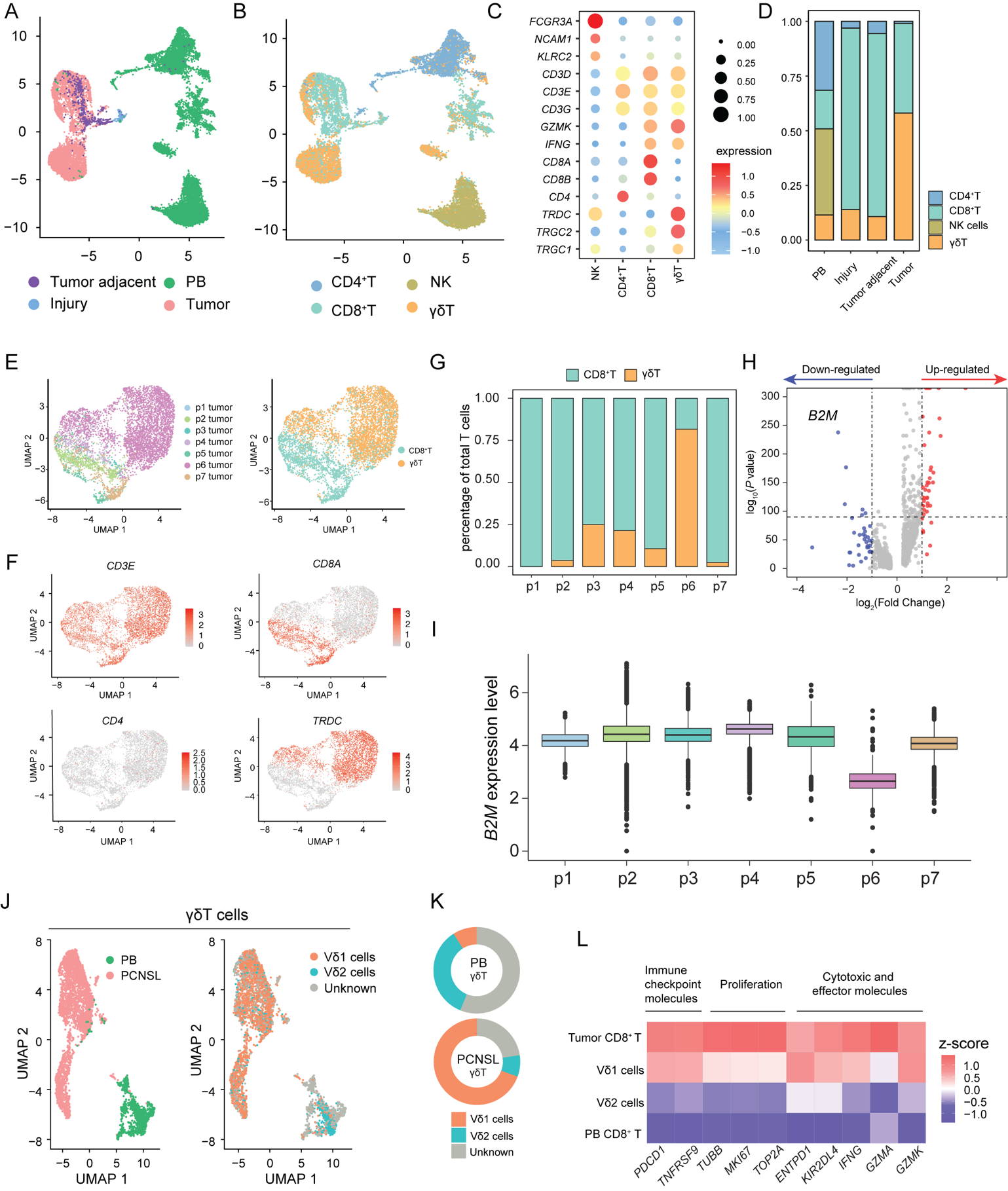
**

**Supplementary Figure 6. Characterization of T/NK cells in PCNSL and non-tumor controls**

(A-B) UMAP visualization of T and NK cells derived from PCNSL tumor samples from 7 patients, tumor-adjacent samples from 3 patients, PB samples from 3 patients, and injured brain samples from 2 patients. Each dot represents an individual cell. Cells are colored according to sample origins (A) and T/NK cell subtypes (B).

(C) Expression levels of selected marker genes across T/NK cell subtypes, including NK cells, CD4^+^ T cells, CD8^+^ T cells, and γδ T cells.

(D) Proportions of CD4^+^ T cells, CD8^+^ T cells, γδ T cells, and NK cells among total T/NK population in PCNSL tumor, tumor-adjacent, PB, and injured-brain samples.

(E) UMAP visualization of T cells from PCNSL tumor samples from 7 patients, colored by patient identities (left) and major T cell subtypes (right).

(F) UMAP plots showing expression levels of selected marker genes: *CD3D* (pan-T cell marker), *CD8A* (CD8^+^ T cells), *CD4* (CD4^+^ T cells), and *TRDC* (γδ T cells).

(G) Proportions of CD8^+^ T cells and γδ T cells among the total CD8^+^ T and γδ T cells in each PCNSL patient.

(H) Volcano plot showing differentially expressed genes in tumor B cells between patient 6 (with the highest percentage of γδ T cell infiltration) and other patients.

(I) *B2M* expression levels in tumor B cells across all PCNSL patients.

(J) UMAP visualization of γδ T cells derived from PCNSL tumor samples from 6 patients with detected γδ T cells and PB samples from 3 patients. Cells are colored according to sample origins (left) and γδ T cell subtypes (right).

(K) Distribution of different γδ T cell subtypes in PB (top) and PCNSL tumor (bottom) samples.

(L) Heatmap showing z-score normalized expression of immune checkpoint genes, proliferation markers, and cytotoxic/effector molecules across T cell subtypes, including CD8^+^ T cells, Vδ1 T cells, Vδ2 T cells, and PB CD8^+^ T cells.

**
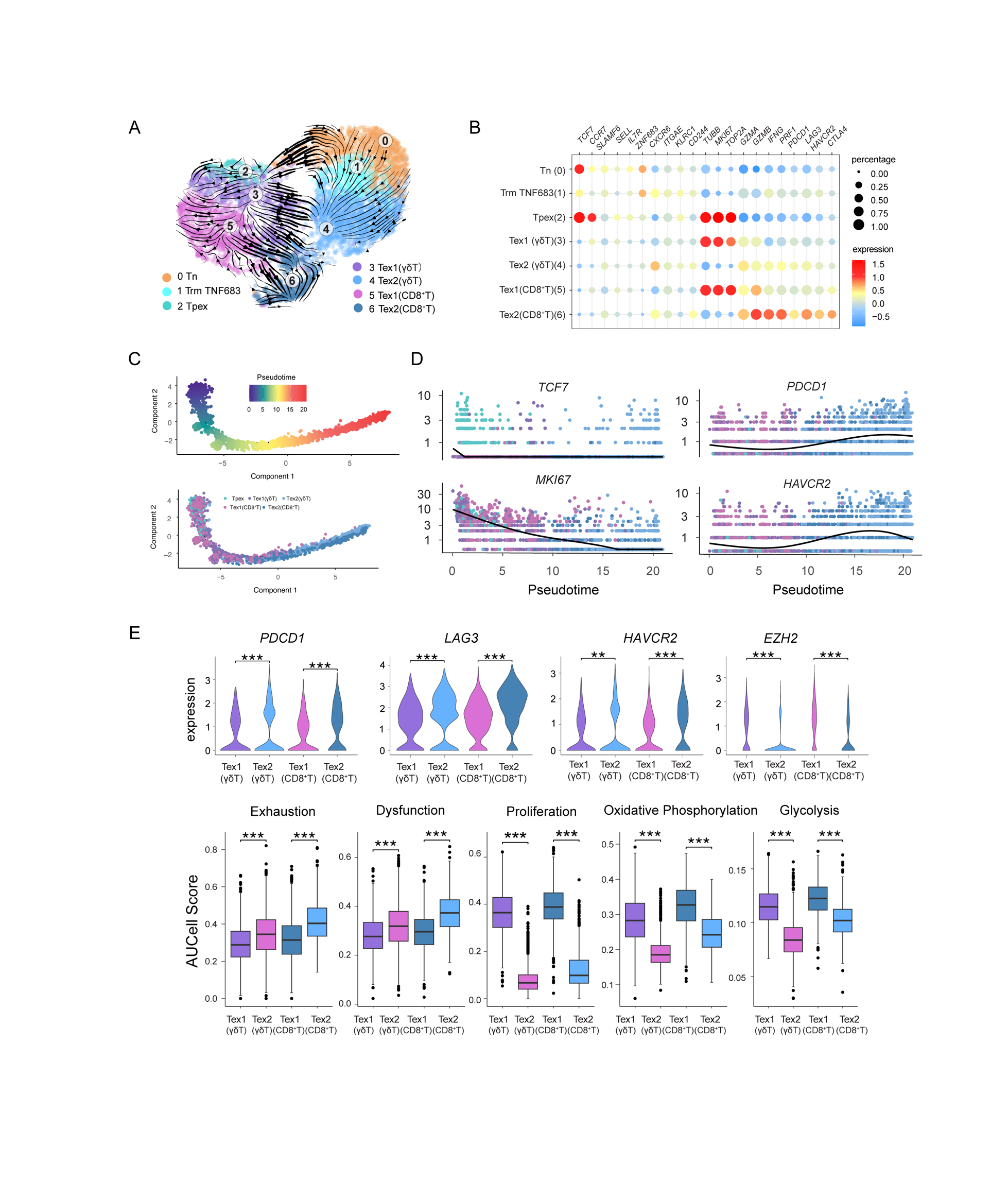
**

**Supplementary Figure 7. Characterization of T-cell exhaustion in PCNSL**

(A) RNA velocity fields overlaid on UMAP, showing the inferred transcriptional dynamics and state transitions from naïve to exhausted states in CD8^+^ and γδ T cells.

(B) Expression levels of selected marker genes across subtypes of CD8^+^ and γδ T cells.

(C) Pseudotime trajectory of T cells constructed using Monocle2. Components 1 and 2 are shown, with each dot representing a single cell. Dots are colored by pseudotime (top) or T cell subtypes (bottom).

(D) Expression dynamics of selected genes along the pseudotime trajectory. Each dot represents an individual cell, colored by T cell subtypes.

(E) Violin plots (top) showing expression levels of selected genes, and box plots (bottom) displaying gene signature scores in Tex1 and Tex2 subsets of CD8^+^ and γδ T cells. Statistical significance of comparisons between groups were assessed determined using two-sided Student’s t-tests (***p* < 0.01, ****p* < 0.001).

**
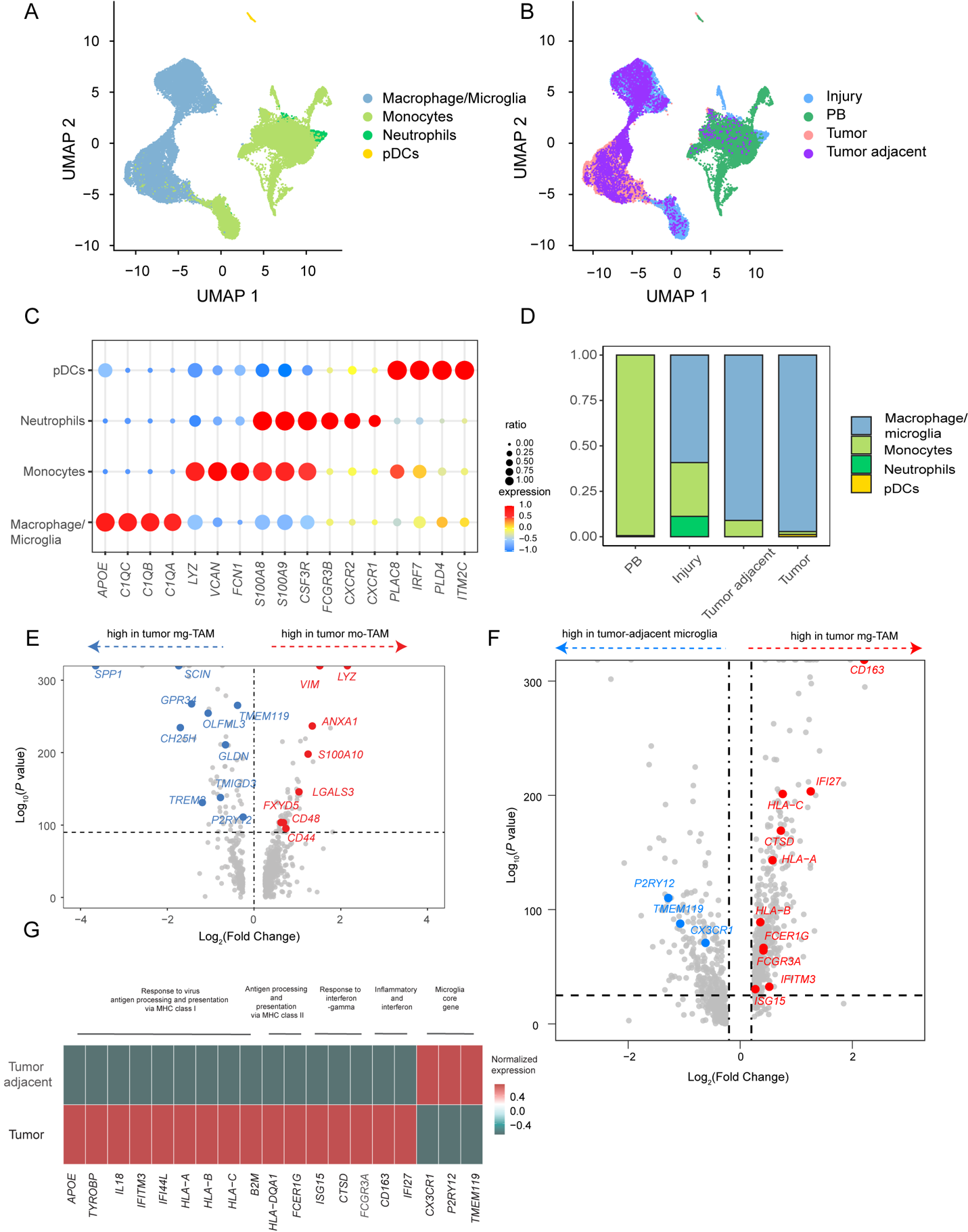
**

**Supplementary Figure 8. Myeloid populations in PCNSL tumor and tumor-adjacent samples**

(A-B) UMAP visualization of myeloid cells derived from PCNSL tumor samples from 7 patients, tumor-adjacent samples from 3 patients, PB samples from 3 patients, and injured brain samples from 2 patients. Each dot represents a single cell, colored by cell types (A) and sample origins (B).

(C) Expression levels of selected marker genes across myeloid cell subtypes.

(D) Proportions of myeloid cell subtypes among total myeloid cells in PCNSL tumor samples, tumor-adjacent tissues, PB samples, and injured brain tissues.

(E) Volcano plots showing differentially expressed genes between microglia-derived tumor-associated macrophages (mg-TAMs) and monocyte-derived tumor-associated macrophages (mo-TAMs) in PCNSL tumor samples.

(F) Volcano plots showing differentially expressed genes between PCNSL mg-TAMs tumors and tumor-adjacent microglia.

(G) Heatmap showing normalized expression of genes related to the indicated biological processes in microglia/mg-TAMs from tumor-adjacent tissues and PCNSL tumor samples.

**
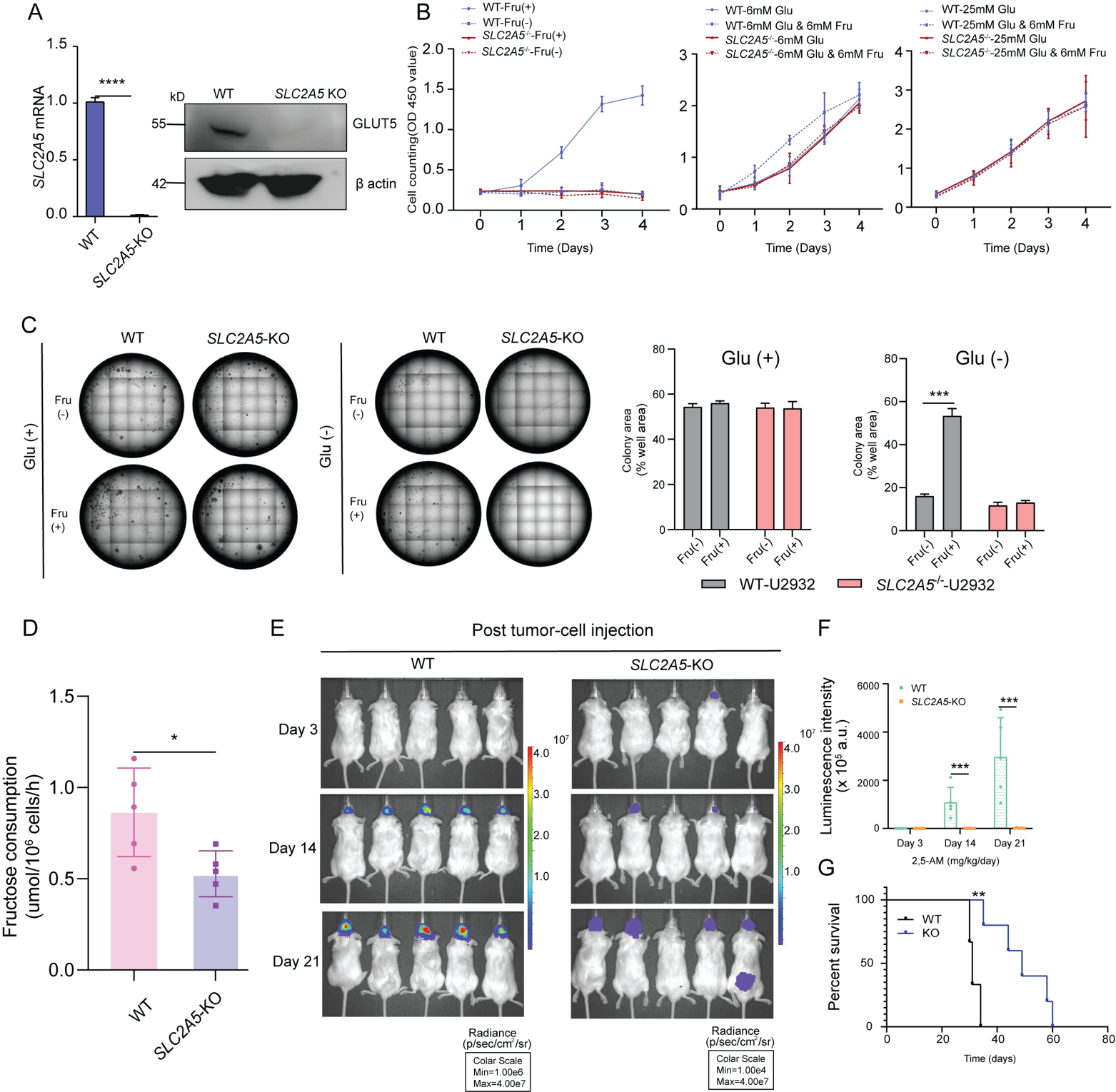
**

**Supplementary Figure 9. *SLC2A5* KO suppresses lymohoma growth under low-glucose conditions**

(A) *SLC2A5* and GLUT5 expression levels in U-2932 wild-type (WT) and *SLC2A5*-knockout cells quantified by PCR (left) and immunoblotting (right) using the indicated antibody.

(B) Cell viability of U-2932 cells with or without *SLC2A5* knockout cultured in RPMI 1640 medium with the indicated glucose and fructose concentrations for 4 days.

(C) Colony formation of U-2932 cells with or without *SLC2A5* knockout cultured in MethoCultTM H4230 medium under glucose-deprived (0 mM) or glucose-sufficient (6 mM) conditions, with (6 mM) or without fructose supplementation, for 14 days.

(D) WT and *SLC2A5*-knockout U-2932 cells were cultured in glucose-free medium for 18 h and then incubated with 10 mM fructose for an additional 18 h. The conditioned media were subsequently collected for the analysis of fructose consumption. Data are presented as mean ± SD, n=5. Asterisks indicate statistical significance (**p* < 0.05).

(E) Luciferase-expressing U-2932 cells with or without *SLC2A5* knockout were intracranially injected into immunodeficient mice (n = 5 per group). Luminescence derived from injected cells was measured on days 3 and 21 post-injection, and relative luminescence intensity is shown (a.u., arbitrary units).

(F) Bar plot summarizing relative luminescence intensity at different time points post tumor-cell injection in mice injected with U-2932 wild-type cells or U-2932 *SLC2A5*-knockout cells. Data represent mean ± SEM for each group (n = 5), with comparisons made between WT and *SLC2A5* knockout groups at each time point. Asterisks indicate statistical significance (****p* < 0.001).

(G) Kaplan–Meier survival analysis of immunodeficient mice (n = 10) intracranially injected with luciferase-expressing U-2932 cells with or without *SLC2A5* knockout.

**Supplementary Tables**

**Supplementary Table 1. Patient and sample information**

Detailed clinical and experimental information, including patient sex, age, diagnosis, disease subtype, tissue source, and sequencing strategy.

**Supplementary Table 2. BCR clonotype sequences and frequencies**

Detailed information of B-cell receptor (BCR) chains, including amino acid sequences, gene usage, nucleotide sequences and the distribution of clonal types.

**Supplementary Table 3. Gene lists for signatures used in this study**

Gene sets corresponding to the signatures of oxidative phosphorylation, glycolysis, exhaustion, dysfunction, and proliferation.
